# Engineering Adenine Deaminase TadA for Precise and PAM‐Flexible Point Mutagenesis and Gradient‐Tuning Endogenous Protein Design

**DOI:** 10.1002/advs.202506644

**Published:** 2025-06-20

**Authors:** Kangli Sun, Si Cheng, Nan Chai, Jianing Mi, Ruixiang Zhang, Qian Qian, Zhiye Zheng, Ke Chen, Dongchang Zeng, Xin Peng, Mengyuan Shen, Degui Zhou, Qinlong Zhu, Qi Liu, Jiantao Tan

**Affiliations:** ^1^ Rice Research Institute Guangdong Academy of Agricultural Sciences Key Laboratory of Genetics and Breeding of High‐Quality Rice in Southern China (Co‐construction by Ministry and Province) Ministry of Agriculture and Rural Affairs Guangdong Key Laboratory of Rice Science and Technology Guangdong Rice Engineering Laboratory Guangzhou 510640 China; ^2^ Guangdong Basic Research Center of Excellence for Precise Breeding of Future Crops State Key Laboratory for Conservation and Utilization of Subtropical Agro‐Bioresources College of Agriculture South China Agricultural University Guangzhou 510642 China; ^3^ Chinese Medicine Guangdong Laboratory Zhuhai 529031 China; ^4^ Guangxi Key Laboratory of Landscape Resources Conservation and Sustainable Utilization in Lijiang River Basin, University Engineering Research Center of Bioinformation and Genetic Improvement of Specialty Crops Guangxi Normal University Guilin 541006 China

**Keywords:** Badh2, gradient‐tuned protein design, point mutagenesis, precise base editing, TadA variants

## Abstract

Base editing enables precise nucleotide substitutions within a relatively broad editing window (5–6 nucleotides). However, considerable bystander editing significantly compromise its accuracy. Point mutagenesis, a powerful approach for gradient‐tuning protein function, facilitates the generation of diverse plant phenotypes to meet the demands of complex environments and consumer preferences. Here, a series of plant base editors is engineered by fusing three optimized TadA8e variants, TadA9, TadA‐LM, and TadA‐dual, with a PAM‐flexible SpRY nickase (SpRYn, with 5′‐NNN PAM recognition). These editors enable A‐to‐G, C‐to‐T, and dual‐base (simultaneous A‐to‐G and C‐to‐T) conversions within a highly condensed active window (1–3 nucleotides). Performance evaluations reveal that the TadDBE (TadA Dual‐Base Editor) achieves the most robust outcomes, delivering dual‐base editing efficiencies ranging from 2.3% to 61.4%, while maintaining minimal off‐target activity. Utilizing TadDBE, targeted point mutagenesis is performed on *OsBadh2*, a gene encoding betaine aldehyde dehydrogenase that plays a critical role in the biosynthesis of 2‐acetyl‐1‐pyrroline (2‐AP), a key aromatic compound. This approach yields rice lines exhibiting gradient‐tuned aromatic profiles and optimized levels of 2‐AP and γ‐aminobutyric acid (GABA). These evolved TadA‐derived editors provide a precise, PAM‐flexible platform for base editing and represent a versatile strategy for generating genome‐edited plants with gradient‐tuned agronomic traits.

## Graphical abstract

Base editing enables precise nucleotide substitutions but limited by bystander editing. This study engineers plant base editors by fusing optimized TadA variants with PAM‐flexible SpRY nickase, enabling A‐to‐G, C‐to‐T, and dual‐base conversions in a highly condensed window (≤3 nucleotides). Additionally, TadDBE (TadA Dual‐Base Editor)‐mediated directed evolution in OsBadh2 generats gradient‐tuned 2‐AP and GABA contents in rice, offering a precise and effective platform for crop breeding.

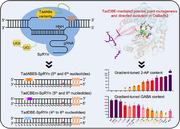

## 1 Introductions

Base editing is a precise and convenient genome editing approach that generates site‐specific base conversions without the need for DNA double‐strand breaks or exogenous donor templates^.[^ ^1^ ^]^ CRISPR/Cas9‐derived base editors (BEs) typically consist of Cas9 nickase (Cas9n, harboring a D10A mutation) fused to various DNA deaminases or glycosylases.^[^ ^2^, ^3^, ^4^, ^5^, ^6^ ^]^ The two primary classes of BEs are cytosine BEs (CBEs) and adenine BEs (ABEs), which mediate C‐to‐T and A‐to‐G conversions, respectively.^[^ ^7^, ^8^ ^]^ CBEs utilize engineered cytidine deaminases, such as PmCDA1/hAID/hAPOBEC/FERNY, in combination with an uracil glycosylase inhibitor (UGI) to generate C‐to‐T transitions.^[^ ^6^, ^9^, ^10^, ^11^ ^]^ However, these editors often produce unintended outcomes, including C‐to‐G and C‐to‐A conversions, or insertions‐deletions (InDels).^[^ ^8^ ^]^ ABEs achieve highly efficient and precise A‐to‐G substitutions, with purity exceeding 99.9%, and minimal InDel frequency by employing re‐evolved TadA8e deaminases.^[^ ^12^ ^]^ Application of ABEs/CBEs is sometimes limited due to their single mutation products. Dual‐base editors (DBEs) enable dual‐base (simultaneous C‐to‐T and A‐to‐G) editing for the directed evolution and saturation mutagenesis with multiple heterogeneous nucleotide substitutions in crops.^[^ ^13^, ^14^, ^15^, ^16^ ^]^ Currently, DBEs available in plants generally require the use of both cytidine and adenine deaminases simultaneously, including notable STEME,^[^ ^14^ ^]^ pDuBE1,^[^ ^17^ ^]^ STCBE‐2,^[^ ^18^ ^]^ SWISS,^[^ ^15^ ^]^ and MoBE^[^ ^16^ ^]^ systems. However, large complex sizes and complicated constructions of these DBEs hinder DNA‐free ribonucleoprotein (RNP) delivery for transgene‐free base editing in plants.^[^ ^19^ ^]^ Moreover, the fusion position and order of deaminases, and competition within the editable window compromise their precision.^[^ ^14^, ^20^ ^]^

Despite their efficiency, existing BE systems exhibit high editing activity within a relatively broad editing window (typically positions M_3_–M_8_ relative to the protospacer adjacent motif (PAM) [at positions, 21–23] where M = A or C).^[^ ^3^, ^10^, ^11^, ^21^ ^]^ This wide activity range often leads to bystander editing and unwanted base changes when multiple cytosines or adenines are present closely, which compromise their precision. Although prime editing provides high precision and minimum byproducts, its unstable editing efficiency in plants and complex design make it unable to replace BEs in a short period of time.^[^ ^1^, ^22^ ^]^ The trade‐off between editing efficiency and precision is a critical technical bottleneck in plant base editing. The original *Streptococcus pyogenes*‐derived SpCas9 recognizes canonical 5′‐NGG PAM, which significantly restricts the applicability of precise BEs with narrow editing windows. This limitation poses substantial challenges for the selection and design of target sites in diverse genomic contexts.^[^ ^22^ ^]^ To overcome these constraints, various Cas9 variants with expanded or flexible PAM recognition capabilities have been engineered, including SpCas9‐NRRH, SpCas9‐NG, SpG, SpRY, ScCas9, SaCas9, and CjCas9.^[^ ^3^, ^5^, ^23^, ^24^, ^25^, ^26^, ^27^ ^]^ However, PAM‐flexible Cas9 variants may induce potential T‐DNA self‐editing and sgRNA‐dependent off‐target effects.^[^ ^13^, ^21^, ^28^ ^]^ Furthermore, highly active cytidine and adenine deaminases induce potential genome‐wide off‐target effects in plant genome and transcriptome.^[^ ^29^, ^30^ ^]^ Thus, PAM restrictions and off‐target risks are limitations of existing BEs in plants as well. Recent advancements have focused on re‐engineering TadA8e to enable precise A‐to‐G, A‐to‐Y, C‐to‐T, C‐to‐G, and dual‐base editing in mammalian cells^[^ ^31^, ^32^, ^33^, ^34^ ^]^ and plants.^[^ ^13^, ^35^ ^]^ They provide similar editing activities and editing windows (5 nt, M_4_–M_8_) for both C‐to‐T and A‐to‐G editing by utilizing a single deaminase, enabling precise dual‐base editing in rice.^[^ ^13^, ^35^ ^]^

Gradient up‐tuning or down‐tuning of protein function can drive the development of diverse plant phenotypes, which is critical for improving crop traits while balancing complex tradeoffs associated with gene pleiotrophy.^[^ ^36^ ^]^ Gradient‐tuned traits are particularly advantageous for screening crop germplasm tailored to different growth environments and consumer preferences, such as variations in plant height, flowering time, amylose content, and aroma. There are differences in the acceptance and consumption of aromatic rice (*Oryza sativa* L.) among countries and regions,^[^ ^37^ ^]^ which may be attributed to the diverse aroma concentrations in cultivars. The compound 2‐acetyl‐1‐pyrroline (2‐AP) is the primary contributor to the distinctive fragrance of aromatic rice, and its biosynthesis is closely linked to the activity of betaine aldehyde dehydrogenase 2 (OsBadh2).^[^ ^38^, ^39^ ^]^ A reduction or loss of OsBadh2 activity transforms nonaromatic rice into aromatic varieties by blocking the conversion of γ‐amino butyraldehyde (GABald) to γ‐amino butyric acid (GABA), leading to the accumulation of 2‐AP.^[^ ^40^, ^41^, ^42^, ^43^ ^]^ However, GABA plays vital roles in physiological processes such as regulating movement, blood pressure, heart rate, and pain perception.^[^ ^44^ ^]^ Through rational design and modification of coding sequences, base editing‐ and prime editing‐mediated point mutagenesis and endogenous protein engineering had been applied in improving herbicide resistance^[^ ^14^, ^45^ ^]^ and increasing CoQ_10_ content^[^ ^46^ ^]^ in rice. Thus, precisely editing OsBadh2 to achieve functionally gradient‐tuned activity can balance 2‐AP and GABA levels in rice. This approach offers a versatile strategy for breeding crops with optimized complex traits to meet diverse agricultural and consumer demands.

In our previous work, we developed multiple high‐efficiency plant BEs, including PhieCBEs,^[^ ^11^ ^]^ PhieABEs,^[^ ^21^ ^]^ and PhieDBEs,^[^ ^20^ ^]^ which demonstrated broad activity windows and flexible PAM recognition. However, these editors were often limited by issues such as bystander editing and unintended byproducts, leading to reduced base editing accuracy. In this study, we engineered the adenine deaminase TadA8e to enable precise A‐to‐G, C‐to‐T, and dual‐base editing within a condensed active window of 1–3 nucleotides in rice. By fusing these TadA variants with the PAM‐flexible SpRY nickase (SpRYn), we systematically evaluated their precision, efficiency, and PAM flexibility across multiple rice endogenous genes. Furthermore, we applied TadDBE (TadA Dual‐Base Editor) to design and execute point mutagenesis‐mediated gradient tuning of rice aroma and GABA content by introducing precise and diverse amino acid mutations in OsBadh2. Consistent with 2‐AP concentration analyses, most *OsBadh2* mutants exhibited significantly increased binding affinity (Δ*G*), highlighting the functional impact of these mutations. Our study introduces evolved TadA‐derived BEs tailored for precise genome editing in plants and presents a universal approach for gradient tuning of protein functions and crop traits.

## 2 Results

### 2.1 TadA‐Derived Editors Enable Precise A‐to‐G and/or C‐to‐T Conversions with SpRYn

To achieve precise base editing in plants, we engineered three rice codon‐optimized TadA variants, TadA9,^[^ ^31^ ^]^ TadA‐LM,^[^ ^32^ ^]^ and TadA‐dual^[^ ^33^ ^]^ (Figure S1A, Supporting Information). Compared with original TadA8e, the TadA9, TadA‐LM, and TadA‐dual were constructed by introducing three (V106W, N108Q, L145T), three (N46L, A48M, V106W), and six (R26G, V28A, A48R, Y73S, H96N, V106W) amino acid substitutions, respectively (Figure S2A, Supporting Information). Using AlphaFold,^[^ ^47^ ^]^ we predicted the crystal structure of these TadA variants and observed that the introduced mutations either established novel hydrogen bonds with adjacent residues or disrupted existing connections (Figure S2B–D, Supporting Information), which may alter the specific binding of TadA variants to their substrate.

**Figure 1:**
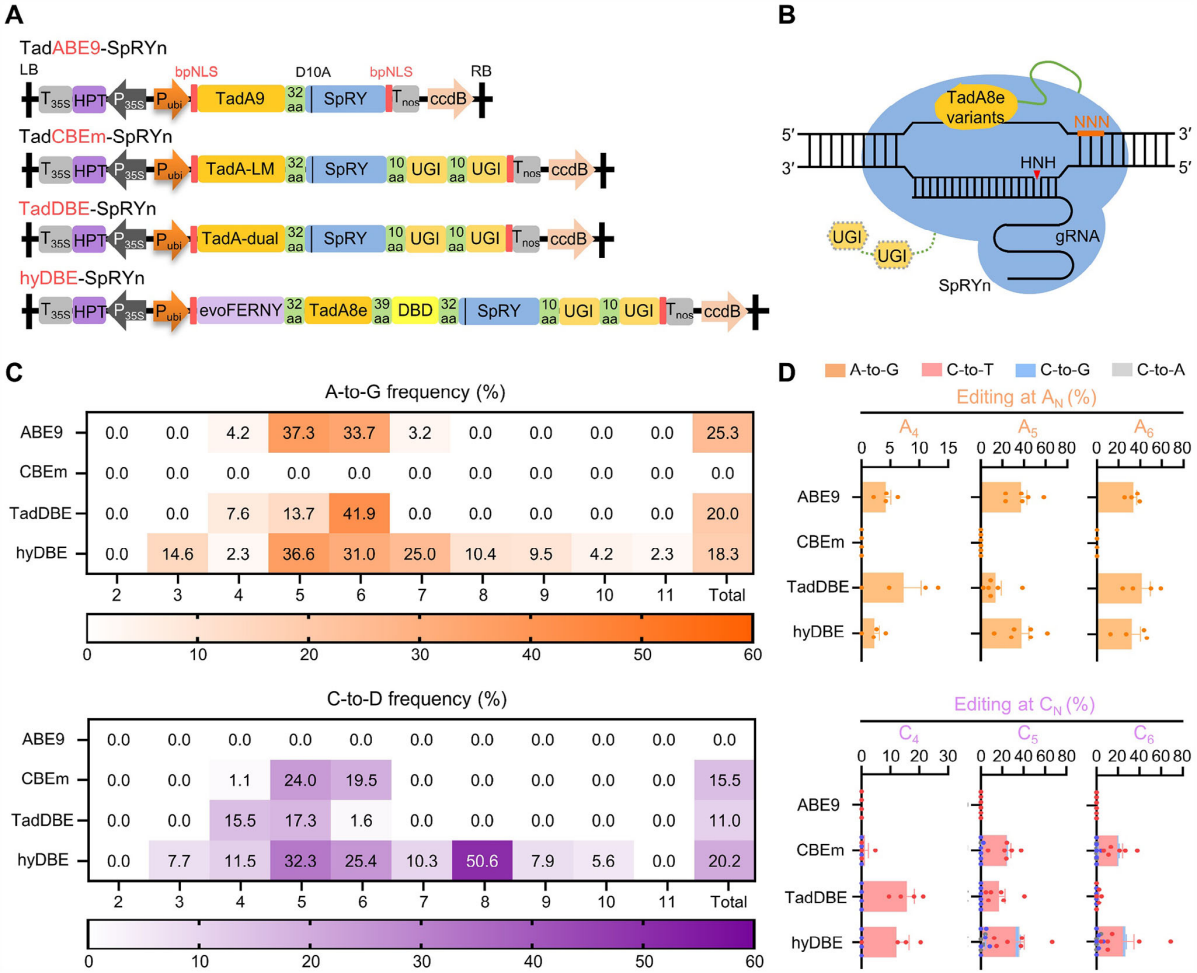
Precise base editing in rice using engineered TadA8e variants. A) Schematic illustrations of TadA‐derived base editors (BEs) in plants. Three engineered TadA variants, TadA9, TadA‐LM, and TadA‐dual, were fused with the PAM‐flexible SpRY nickase (D10A). A dual‐base editor hyDBE containing the cytosine deaminase evoFERNY and the adenine deaminase TadA8e was used as a control check. bpNLS, bipartite nuclear localization sequence; UGI, uracil glycosylase inhibitor; 32, 39, and 10 aa, 32, 39, and 10 amino acid linkers. B) Illustration of the working model of TadA‐derived BEs. C) Heatmaps illustrating the A‐to‐G and C‐to‐D editing efficiencies and active windows of ABE9, CBEm, TadDBE, and hyDBE at 13 endogenous target sites in rice. Average editing frequencies from the 2^nd^ to the 11^th^ nucleotides within the editing windows are analyzed separately. The total editing efficiencies within the editing windows for each BE were calculated by using the following equation: A‐to‐G frequency = *N_1_* × *N_3_* ^−1^ × 2^−1^ and C‐to‐D frequency = *N_2_* × *N_3_* ^−1^ × 2^−1^. *N_1_*: number of alleles with A‐to‐G edits; *N_2_*: number of alleles with C‐to‐D edits; *N_3_*: total number of transgenic positive calli. D = T, G or A. D) Base conversion efficiencies and types of each editor at the key focus M_4_–M_6_ nucleotides. M = A or C.

Precise BEs are often constrained by narrow editing windows and PAM recognition motifs, which restrict their ability to target specific bases effectively. To address these limitations, we fused the three engineered TadA variants at the N‐terminus of the nearly PAM‐free SpRY nickase, generating TadABE9‐SpRYn, TadCBEm‐SpRYn and TadDBE‐SpRYn BEs. For TadCBEm‐SpRYn and TadDBE‐SpRYn, the inclusion of an Uracil Glycosylase Inhibitor at the C‐terminus of SpRYn was essential to facilitate effective cytosine base editing (**Figure** **1**A,B).

**Figure 2:**
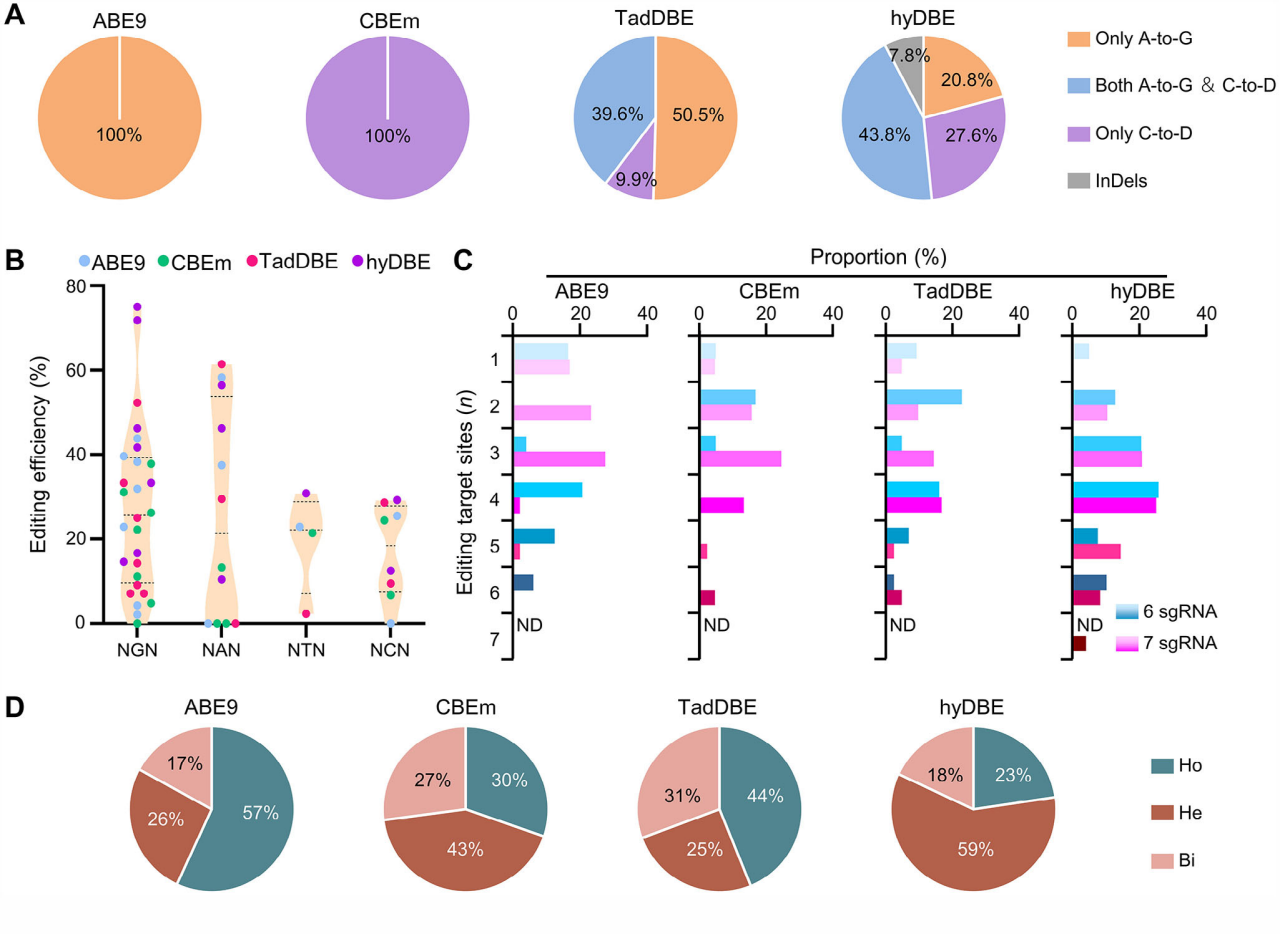
A) TadA‐derived BEs enable multiplex genome editing with PAM‐flexible target recognition. Proportions of editing outcomes generated by ABE9, CBEm, TadDBE, and hyDBE within the targeted range. B) Editing efficiencies at 5′‐NGN, 5′‐NAN, 5′‐NTN, and 5′‐NCN PAM targets calculated for all SpRYn‐based edited rice calli. C) Proportion of multi‐target sites edited simultaneously by each editor using 6 and 7 sgRNA cassettes in transgenic calli. ND, not detectable. D) Proportion of mutation types induced by each editor across all tested target sites. Ho, homozygous; He, heterozygous; Bi, biallelic mutations.

To evaluate the editing efficiency and specificity of the TadA‐derived editors in plants, 13 sgRNAs with 5′‐NNN PAMs were designed to target 12 endogenous rice genes (Table S1, Supporting Information). These target sites were selected to include multiple adenines and/or cytosines, enabling comprehensive assessment of the editors' base‐editing profiles. In transgenic rice calli, the TadABE9‐SpRYn (abbreviated as ABE9) achieved an average A‐to‐G editing efficiency of 25.3% within a narrow editing window, primarily spanning two nucleotides (37.3% at A_5_ and 33.7% at A_6_) (Figure 1C,D). In contrast, the TadCBEm‐SpRYn editor (abbreviated as CBEm) mediated specific C‐to‐T (average of 15.1% ratio) and C‐to‐G (average of 0.4% ratio) conversions, with editing efficiencies of 24.0% and 19.5% at C_5_ and C_6_, respectively, while exhibiting no adenine base editing activity and without InDel products (Figures 1C,D and **2**A). These findings indicate that the engineered TadA9 and TadA‐LM variants significantly improved editing precision by narrowing the active window. However, this improvement in specificity was accompanied by a partial or complete loss of adenine base‐editing activity.

In our recent study,^[^ ^20^ ^]^ the C‐A‐D‐SpGn from the PhieDBE toolbox, which integrates cytidine deaminase evoFERNY, adenosine deaminase TadA8e, and single‐stranded DNA‐binding domain (DBD), demonstrated high‐efficiency dual‐base editing activity within a broad editing window (A_3_, M_5_–M_9_). Building on this, we replaced SpGn in C‐A‐D‐SpGn with SpRYn, creating hyDBE‐SpRYn (abbreviated as hyDBE) to serve as a control check‌ (CK) alongside TadDBE‐SpRYn (abbreviated as TadDBE) (Figure 1A). The A‐to‐G and C‐to‐D (D = T, G, or A) editing efficiencies of hyDBE ranged from 2.3% to 36.6% and 5.6% to 50.6% (average of 17.7% for C‐to‐T, 1.8% for C‐to‐G, and 0.7% for C‐to‐A editing), respectively, with edits distributed across M_3_–M_10_ and A_11_ (Figure 1C). Using the same sgRNAs, TadDBE produced slightly higher average A‐to‐G editing efficiencies (20.0% vs 18.3%) but lower average C‐to‐D editing efficiencies (11.0% vs 20.2%) compared to hyDBE (Figure 1C). Consistent with findings from recent studies,^[^ ^13^, ^35^ ^]^ structure‐guided engineering of TadA‐dual reduced both adenine and cytosine bystander editing, refining the editing window to three nucleotides (M_4_–M_6_). Within this window, TadDBE exhibited an increasing A‐to‐G editing activity trend closer to the PAM, with the highest efficiency observed at A_6_ (41.9%). C‐to‐T edits were predominantly located at C_4_ (15.5%) and C_5_ (17.3%) (Figure 1C,D). Furthermore, the purity of edits generated by TadDBE was notably higher than those from hyDBE. TadDBE exclusively performed A‐to‐G (100%) and C‐to‐T (100%) edits, while hyDBE edits included A‐to‐G (100%), C‐to‐T (90.4%), C‐to‐G (1.4%), C‐to‐A (0.4%), and a small percentage of InDels (7.8%) (Figures 1D and 2A). TadDBE primarily facilitated A‐to‐G (50.5%) and dual‐base (39.6%) conversions, with a minor proportion of C‐to‐T conversions (9.9%) (Figure 2A). These results reflect the reliance of TadDBE on a single TadA deaminase. In contrast, hyDBE induced 43.8% dual‐base conversions, 27.6% C‐to‐D conversions, 20.8% A‐to‐G conversions, and 7.8% InDels (Figure 2A), attributed to the active role of evoFERNY. Collectively, these data demonstrate that TadA‐derived BEs are highly efficient at generating precise edits while sacrificing some editing efficiency, enabling A‐to‐G, C‐to‐T, or dual‐base conversions within a narrow editing window while minimizing byproducts.

### 2.2 TadA‐Derived Editors Induce PAM‐Flexible Genome Editing in Plants

To assess the versatility of TadA‐derived editors, we analyzed the PAM recognition preferences of various BE constructs. When fused with SpRYn, all four BEs demonstrated robust editing activities at target sites with 5′‐NRN PAM (R = G or A). In contrast, relatively weaker activities were observed at targets with 5′‐NYN PAM (Y = C or T) (Figure 2B). These findings confirmed the compatibility of TadA‐derived variants with the PAM‐flexible SpRYn.

To assess the potential of TadA‐derived editors for multiplex genome editing, two groups of test loci were designed for each BE. One group contained six 5′‐NNN PAM targets, and the other contained seven (Table S1, Supporting Information). For ABE9 and CBEm, 37.8% and 29.4% of transgenic calli, respectively, exhibited simultaneous mutations at three or more target sites. However, only 3.1% and 2.2% of events contained mutations at six or more loci simultaneously (Figure 2C; Table S2, Supporting Information). In comparison, hyDBE achieved a significantly higher average mutant percentage (68.3%) in calli with at least three edited sites, whereas TadDBE demonstrated a lower average percentage of 33.8% (Figure 2C; Table S2, Supporting Information). These results suggest that hyDBE is the most effective construct among those tested for multiplex genome editing. Beyond generating heterozygous mutations, homozygous and biallelic mutations are particularly desirable for their feasibility in T_0_ genotyping and predictable inheritance patterns in subsequent generations. We analyzed the mutation types induced by the various BEs across 13 target sites. An average of 57–75% of the edited calli produced by TadA‐derived editors, particularly TadDBE (44% homozygous and 31% biallelic mutations), exhibited homozygous or biallelic mutations. In contrast, only 41% of hyDBE lines showed similar mutation types (Figure 2D; Table S3, Supporting Information). Furthermore, TadA‐derived editors exhibited a degree of sequence context preference at 5′‐CA motif (the editing site is underlined), while no obvious cytosine base preference was observed at the 13 tested target (Figure S3, Supporting Information).

PAM‐flexible Cas9 variants primarily induce potential T‐DNA self‐editing and sgRNA‐dependent off‐target effects, raising significant public concerns about genome editing safety.^[^ ^13^, ^21^, ^28^ ^]^ To evaluate self‐editing effects induced by newly developed editors, we PCR amplified and sequenced the sgRNA cassettes in all transgenic calli for TS1–TS13, and no self‐editing events were detected in ABE9, CBEm, TadDBE, and hyDBE‐mediated editing (Figure S4, Supporting Information). Next, we evaluated effects of the TadA‐derived editors and hyDBE by examining all potential off‐target loci (within up to two nucleotide mismatches). Consistent with a recent study,^[^ ^13^ ^]^ our results indicated that TadA‐derived editors exhibited minimal sgRNA‐dependent off‐target effects, with only weak activity detected at individual predicted off‐target sites. In contrast, hyDBE showed off‐target editing at multiple loci, with the highest observed frequency of 8.3% (Figure S5, Supporting Information). To mitigate sgRNA‐independent off‐target effects induced by deaminases, we introduced a V106W mutation into the TadA‐derived variants, based on its performance for reducing nontargeted activity in mammalian cells.^[^ ^33^ ^]^ Despite there is lack of significant genome‐wide gRNA‐independent off‐target effects by TadA‐derived editors in rice, further validation via whole‐genome and transcriptome sequencing is required to comprehensively assess editing safety.

### 2.3 TadDBE Mediates Precise Point Mutation for Rational Protein Engineering in Rice

Dual BEs significantly broaden the range of achievable amino acid conversions, introducing nine additional types of amino acid changes compared to single BEs (Figure S6A,B, Supporting Information), thereby expanding the potential for precise point mutagenesis research. However, wide active windows and activated cytosine deaminases often result in unexpected bystander edits and undesired nonsense‐codon mutations, such as the V1TS3‐*Chalk5* editing mediated by C‐A‐D‐SpGn.^[^ ^20^ ^]^ It leads to complete loss of protein function, and is not conducive to exploring the contribution of single amino acid residue change to protein function. Given TadDBE′s balance performance across precision and efficiency in dual‐base conversions, it is employed to facilitate rational protein engineering through precise point mutation in rice. In this study, *OsBadh2*, an allele closely related to aroma traits, was targeted for mutagenesis to generate gradient‐tuned functional mutants.

**Figure 3:**
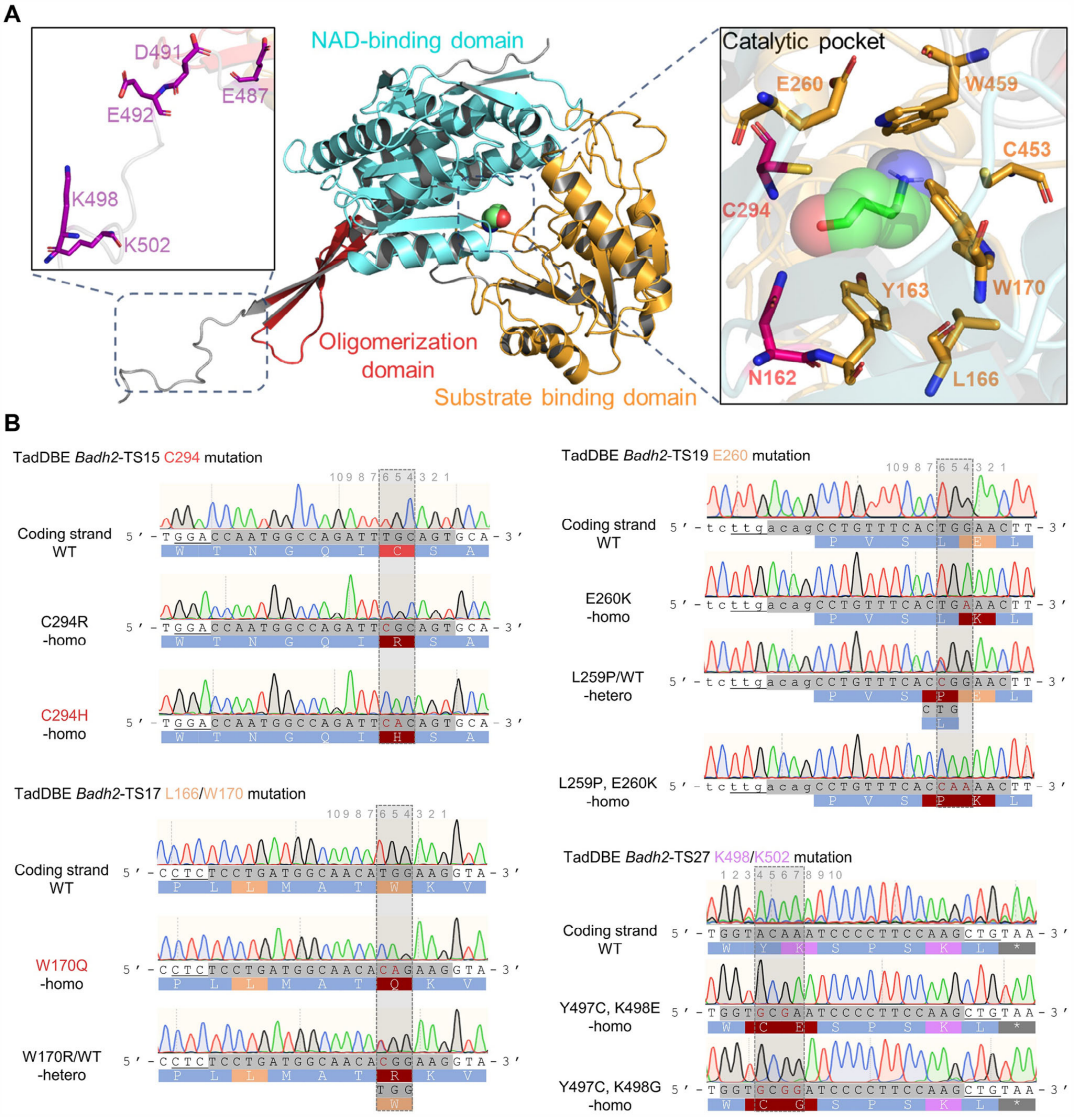
A) Engineering the OsBadh2 by TadDBE‐mediated precise point mutation in rice. 3D structure of the functional domains of OsBadh2. The catalytic (pink), substrate‐binding pocket (orange) and salt‐bridge (purple) residues are annotated. The interaction between GABald and OsBadh2 catalytic pocket is predicted by using AMDock. B) Precise multiple amino acid substitutions were introduced at the *Badh2*‐TS15, ‐TS17, ‐TS19 and ‐TS27 loci by TadDBE in T_0_ plants. The mutated nucleotides and amino acids are highlighted in crimson and crimson background, respectively.

*OsBadh2* encodes betaine aldehyde dehydrogenase, comprising three functional domains: NAD binding, substrate binding, and oligomerization domains.^[^ ^38^ ^]^ Homology‐based structural modeling and analysis identified two residues, N162 and C294, as putative catalytic sites, while six residues—Y163, L166, W170, E260, C453, and W459—were predicted to constitute the substrate‐binding pocket.^[^ ^38^ ^]^ Additionally, five charged residues (E487, D491, E492, K498, and K502) were proposed to form key inter‐subunit salt bridges,^[^ ^48^ ^]^ essential for dimerization (**Figure** **3**A; Figure S1B, Supporting Information). Despite these insights, the atomic‐level interaction between OsBadh2 and its substrate, GABald, remains unresolved. To investigate this interaction, molecular docking analyses were conducted, focusing on Δ*G* values, an important parameter in identifying optimal docking configurations for ligand binding.^[^ ^49^ ^]^ Candidates exhibiting the lowest Δ*G* values were prioritized as potential docking modes. The substrate‐binding pocket of OsBadh2 was modeled with GABald, and the compound with the minimum Δ*G* value (−4.3 kcal·mol^−1^) was selected as the optimal docking configuration (**Table** **1**; Figure S7A, Supporting Information). Further structural analysis utilizing the AlphaFold‐predicted crystal structure of the dimerized form of OsBadh2 revealed several residues at the C‐terminus forming hypothetical hydrogen bonds with residues from adjacent monomers. Among these, the charged interaction between D491 and K277 demonstrated the strongest connectivity, with a bond distance of 2.5 Å (Figure S8, Supporting Information).

**Table 1:** Enzyme activity characterization of OsBadh2 variants and associated 2‐AP and GABA contents in rice grains.

Line No.	Nucleotide mutation	Mutation type	Functional site	ΔΔ*G* (kcal∙mol^−1^)	GABA (mg∙kg^−1^ FW)	2‐AP (µg∙kg^−1^ FW)	Weaking degree
NIP	–	–	Full function	0.00	286.15	0.0	0%
DHX	r: GGTAAAAAGATTATGGCTTC m: GGTATATA——–TTTC	P252*	Loss‐of‐function	–	111.53	262.17	100%
*badh2*	r: AGT‐ACCTCCGCGCAATCGCG m: AGTTACCTCCGCGCAATCGCG	E91*	Loss‐of‐function	–	66.27	89.07	100%
TD^1st^ #2	r: CAGG[AAC]TATCCTCTCCTGA m: CAGG[AGC]TATCCTCTCCTGA	N162S (TS14)	Catalysis	1.15	97.38	84.65	95.0%
	r: CCAATGGCCAGATT[TGC]AGT m: CCAATGGCCAGATT[CAC]AGT	C294H (TS15)	Catalysis				
TD^2nd^ #1	r: TCTACTGCAGGAAC[TAT]CCT m: TCTACTGCAGGAAC[CAT]CCT	Y163H (TS16)	Binding pocket	1.00	168.45	54.27	60.9%
	r: TCCTGATGGCAACA[TGG]AAG m: TCCTGATGGCAACA[CAG]AAG	W170Q (TS17)	Binding pocket				
TD^2nd^ #18	r: TCTACTGCAGGAAC[TAT]CCT m: TCTACTGCAGGAAC[CAT]CCT	Y163H (TS16)	Binding pocket	1.20	143.57	60.38	67.8%
	r: TCCTGATGGCAACA[TGG]AAG m: TCCTGATGGCAACA[CGG]AAG	W170R (TS17)	Binding pocket				
TD^3rd^ #2	r: GAACTATCCTCTCCTGATGG m: GAACTATCCTCTCCTGATGG	WT (TS18)	–	0.22	184.50	20.82	23.4%
	r: ACAGCCTGTTTCA[CTG]GAAC m: ACAGCCTGTTTCA[CCG]GAAC	L259P (TS19)	Unknown				
TD^4th^ #10	r: ACTGCTCGCAACCC[TGC]TTC m: ACTGCTCGCAACCC[CGC]TTC	C453R (TS20)	Binding pocket	2.90	94.39	82.31	92.4%
	r: TCTGCCAAGCTCCA[TGG]GGC m: TCTGCCAAGCTCCA[CGG]GGC	W459R (TS21)	Binding pocket				
TD^4th^ #18	r: ACTGCTCGCAACCC[TGC]TTC m: ACTGCTCGCAACCC[CAC]TTC	C453H (TS20)	Binding pocket	0.40	116.38	64.30	72.2%
	r: TCTGCCAAGCTCCATGGGGC m: TCTGCCAAGCTCCATGGGGC	WT (TS21)	–				
TD^5th^ #13	r: GACG[GAG]TACGCCTCCGATG m: GACG[GGG]TACGCCTCCGATG	E487G (TS22)	Salt bridge	−0.07	269.48	0.0	0%
	r: GTACAAATCCCCTTCCAAGC m: GTACAAATCCCCTTCCAAGC	WT (TS23)	–				
TD^6th^ #2	r: ACGGAGTACGCCTCCGATGA m: ACGGAGTACGCCTCCGATGA	WT (TS24)	–	0.30	204.04	32.53	36.5%
	r: TTCC[AAG]CTGTAATGTAATA m: TTCC[GAG]CTGTAATGTAATA	K502E (TS25)	Salt bridge				
TD^7th^ #2	r: GAGTACGCCTCC[GAT]GAGCC r: GAGTACGCCTCC[GAC]GAGCC	WT (TS26)	–	−0.45	292.44	0.0	0%
	r: GG[TAC][AAA]TCCCCTTCCAAG m: GG[TGC][GGA]TCCCCTTCCAAG	Y497C, K498G (TS27)	Unknown, Salt bridge				
TD^7th^ #11	r: GAGTACGCCTCC[GAT][GAG]CC m: GAGTACGCCTCC[GAC][AAG]CC	E492K (TS26)	Salt bridge	0.29	199.98	28.35	31.8%
	r: GG[TAC][AAA]TCCCCTTCCAAG m: GG[TGC][GAA]TCCCCTTCCAAG	Y497C, K498E (TS27)	Unknown, Salt bridge				
TD^7th^ #18	r: GAGTACGCCTCCGAT[GAG]CC m: GAGTACGCCTCCGAT[AAG]CC	E492K (TS26)	Salt bridge	0.12	203.63	17.63	19.8%
	r: GGTACAAATCCCCTTCCAAG m: GGTACAAATCCCCTTCCAAG	WT (TS27)	–				

*Note*: ΔΔ*G*, binding affinity change upon mutation, defined as ΔΔ*G* = Δ*G*_mutant_ – Δ*G*_WT_. The nucleotide conversions are highlighted in crimson. FW, fresh weight.

To systematically generate gradient‐weakened *OsBadh2* mutants, seven groups of sgRNA‐expression cassettes were designed and assembled into TadDBE constructs, with each group targeting two specific residues (Table S4, Supporting Information). These targets were strategically selected: *Badh2*‐TS14 and TS15 focused on catalytic residues, *Badh2*‐TS16–TS21 targeted residues within the catalytic pocket, and *Badh2*‐TS22–TS27 targeted inter‐subunit bridging residues (Figure 3B; Figure S9, Supporting Information). The nonfragrant *japonica* cultivar Nipponbare (NIP), containing functional OsBadh2, was used as the recipient for *Agrobacterium*‐mediated transformation. In T_0_ plants, amplicon next‐generation sequencing (NGS) revealed precise and high‐efficiency dual‐base editing, producing diverse amino acid mutations at 1–2 target residues (Table S4, Supporting Information). For instance, at *Badh2*‐TS15, TadDBE efficiently induced C294R (T_6_G_5_C_4_>C_6_G_5_C_4_, 40.9% mutagenesis efficiency) and C294H (T_6_G_5_C_4_>C_6_A_5_C_4_, 18.2% mutagenesis efficiency) mutations at the catalytic residue C294 through simultaneous C‐to‐T and A‐to‐G conversion and/or respective base conversions. These edits occurred without introducing unintended changes at adjacent residues (Figure 3B; Table S4, Supporting Information). TadDBE′s precise point mutagenesis was achieved by adjusting the relative positioning of the target sequence within its editing window, leveraging its flexible PAM recognition. Examples include E260K (G_4_A_3_A_2_>A_4_A_3_A_2_), L259P (C_7_T_6_G_5_>C_7_C_6_G_5_), and dual mutations L259P and E260K (C_7_T_6_G_5_G_4_A_3_A_2_>C_7_C_6_A_5_A_4_A_3_A_2_) at *Badh2*‐TS19 (Figure 3B). In addition, TadDBE demonstrated a preferential editing bias toward adenine (e.g., A_4_C_5_A_6_A_7_>G_4_C_5_G_6_G_7_ at *Badh2*‐TS27) over cytosine (Figure 3B), likely due to the inherent adenine deamination activity of the TadA8e variant. Notably, TadDBE‐mediated *OsBadh2* edits generated a substantial number of homozygous mutants in the T_0_ generation (Figure 3B; Figure S9, Supporting Information). This significantly reduced the labor required for isolating stable genetic offspring and shortened the breeding cycle.

### 2.4 Gradient‐Tuning and Balancing of 2‐AP and GABA Contents in Rice

Recent structural analyses by Li et al.^[^ ^50^ ^]^ and Phitaktansakul et al.^[^ ^42^ ^]^ of select OsBadh2 mutants (D65E, Y77*, K244I, A307T, L436F) revealed that alterations in active site structures lead to reduced or null enzyme activity. However, these mutants failed to produce gradient‐tuned rice aroma. To address this, we quantified 2‐AP contents in 11 homozygous T_2_ lines harboring novel *OsBadh2* alleles by gas chromatography–mass spectrometry (GC‐MS), including three lines with residue changes (C294H, W170Q, and C453H) introduced by simultaneous dual‐base conversions (Table 1). CK samples included wild‐type NIP, a frameshift mutant (*badh2*) generated from NIP carrying a premature termination codon (E91*), and the fragrant rice DaoHuaXiang (DHX), which harbors an 8‐bp InDel and three SNPs in exon 7 of *OsBadh2* (Table 1). Quantitative analysis revealed that 2‐AP was present in most mutant lines and DHX, with the latter exhibiting the highest concentration (262.17 µg∙kg^−1^). In contrast, 2‐AP was absent in the wild‐type NIP and two novel *OsBadh2* mutants containing E487G (TD^5th^ #13) and Y497C, K498G (TD^7th^ #2) conversions (Table 1; Figure S10A,B, Supporting Information). Notably, mutants with N162S and C294H substitutions at catalytic residues showed a significant reduction in enzymatic interaction with substrate oxygen. This deficiency resulted in a 2‐AP content in TD^1st^ #2 (84.65 µg∙kg^−1^) that was comparable to that observed in the loss‐of‐function mutant (89.07 µg∙kg^−1^) (Table 1; Figure S7A, Supporting Information). Five lines (TD^2nd^ #1, TD^2nd^ #18, TD^3rd^ #2, TD^4th^ #10, and TD^4th^ #18) with mutations at substrate‐binding residues demonstrated gradient‐increased 2‐AP levels (20.82–82.31 µg∙kg^−1^) and moderate reductions in *OsBadh2* activity (23.4%–92.4%, calculated relative to the 2‐AP concentration in *badh2* mutant) (Table 1). These findings indicate that nonterminating mutations at distinct residue sites variably influence the enzyme′s substrate‐binding affinity. For mutations affecting salt bridges between dimer interfaces, three lines (TD^6th^ #2, TD^7th^ #11, and TD^7th^ #18) produced variants with low reductions in OsBadh2 activity (19.8–36.5%) and weak aroma levels (17.63–32.53 µg∙kg^−1^) (Table 1), suggesting that while hydrogen bonding at the dimer interface contributes to enzyme vitality, its overall impact on enzyme vitality is not significant.

**Figure 4:**
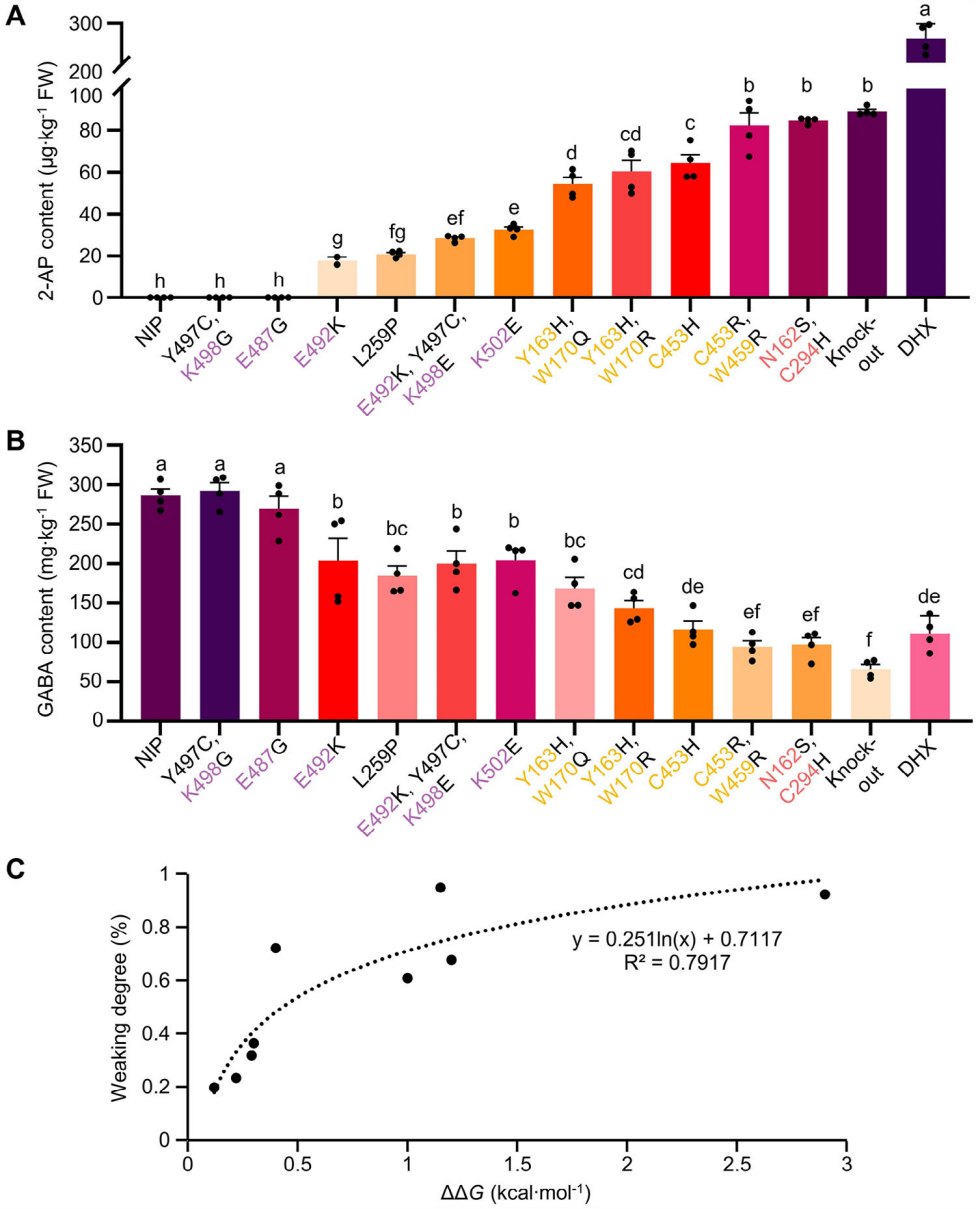
Point mutation‐mediated gradient‐tuning of 2‐AP and GABA contents in rice. **(A–B)** Quantitative assessment of 2‐AP A) and GABA B) contents in Nipponbare (NIP), *badh2* (knock‐out mutant), DaoHuaXiang (DHX, a fragrance rice cultivar) and TadDBE‐mediated point mutants. The catalytic (pink), substrate‐binding pocket (orange), and salt‐bridge (purple) residues are annotated. Means (± SE, *n* = 4) with the same letter are not significantly different, as assessed by Duncan's multiple range test (*P* < 0.05). C) Curve fitting and correlation analysis of binding affinity change (ΔΔ*G*) and the weaking degree of OsBadh2 activity.

Complete loss‐of‐function or weakened activity of *OsBadh2* inevitably disrupts GABA biosynthesis. High‐performance liquid chromatography (HPLC) analysis revealed GABA concentrations ranging from 66.67 to 292.44 mg∙kg^−1^ across the tested lines. TD^7th^ #2 exhibited the highest GABA content (292.44 mg∙kg^−1^), while *badh2* mutant showed the lowest GABA levels (66.67 mg∙kg^−1^) (Table 1; Figure S10C–E, Supporting Information). As anticipated, the concentrations of 2‐AP and GABA displayed an inverse relationship across the tested lines. Notably, an optimal balance was achieved in lines TD^2nd^ #1 (with Y163H and W170Q mutations) and TD^2nd^ #18 (with Y163H and W170R mutations), obtaining 143.58–168.45 µg∙kg^−1^ 2‐AP and 54.27–60.38 mg∙kg^−1^ GABA contents (**Figure** **4**A,B; Table 1). Despite gradient‐tuning 2‐AP and GABA contents, TadDBE‐mediated point mutations exhibited similar yield agronomic traits to wild‐type NIP (Figure S11, Supporting Information), suggesting that precise point mutagenesis‐mediated gradient‐tuning enzyme activity is an effective breeding strategy that does not adversely impact major agronomic traits. Overall, these findings indicate the utility of precise point mutagenesis in gradient‐tuning of endogenous protein functions. By gradient‐tuning OsBadh2 activity, it is possible to engineer an optimal balance between complex traits.

To further investigate the structural effects of *OsBadh2* mutations, we docked GABald to the substrate‐binding pocket of various OsBadh2 variants. Docking analysis revealed that the electron atoms of GABald formed several novel hydrogen bonds with amino acid residues in the substrate‐binding pocket of OsBadh2 variants, while disrupting the original hydrogen bond connections (Figure S7A, Supporting Information). In the TD^2nd^ #1 and #18 lines, the substitution of the nonpolar tryptophan with the polar glutamine (W170Q) and electrically charged arginine (W170R), respectively, resulted in strong electrostatic attraction between the negatively charged aldehyde group of GABald and the positively charged amino groups of Q170 or R170 (Figure S7A, Supporting Information). This observation aligns with the 2‐AP content analysis, where these mutations led to positive changes in binding affinity (ΔΔ*G*) (Table 1), indicating reduced substrate binding affinity. Similarly, in the dual‐residue mutant TD^4th^ #10, incorrect substrate localization was observed, leading to a significant increase in ΔΔ*G* and a corresponding decline in enzyme activity (Table 1; Figure S7A, Supporting Information). Structure characteristic analysis of mutation residues at the dimer interface revealed the formation of multiple novel hydrogen bonds between adjacent monomers in TD^7th^ #2, carrying the Y497C and K498G mutations. This resulted in a more compact dimer structure with a lower binding affinity (ΔΔ*G* = −0.45 kcal·mol^−1^) compared to that of the wild type (WT) enzyme (Table 1; Figure S7B, Supporting Information). In contrast, reversals of charge properties in charged residues, such as K502E in TD^6th^ #2, E492K and K498E in TD7^th^ #11, and E492K in TD7^th^ #18, increased atomic distances between adjacent monomers, including key interactions such as K498–N320 and D491–K277 (Figure S7B, Supporting Information). These alterations were associated with slight increases in ΔΔ*G* (0.12–0.30 kcal·mol^−1^) and mild reductions in enzyme activity (Table 1). To explore the relationship between ΔΔ*G* and enzyme activity, we generated scatter plots and established fitting curves. Among various models tested, logarithmic function modeling provided the best fit, with highest coefficient of determination (*R* ^2^ = 0.7917) (Figure 4C). This result demonstrates the potential for utilizing mathematical models to design endogenous proteins and predict their functional activity.

## 3 Discussion

BEs represent efficient and user‐friendly tools for precise and predictable plant genome editing. Utilizing highly active cytosine (e.g., PmCDA1, A3Bctd, rAPOBEC1, hAPOBEC3A, and evoFERNY) and/or adenine (e.g., TadA8e, TadA9 and TadA9e) deaminase variants (Table S5, Supporting Information), BEs currently available in plants facilitate extensive deamination within a broad activity window (≈6 nucleotides), which may lead to undesired bystander edits and unintended byproducts when multiple adenines or cytosines are present closely. In this study, we evaluated the deaminase activity of three TadA‐derived variants, TadA9,^[^ ^31^ ^]^ TadA‐LM,^[^ ^32^ ^]^ and TadA‐dual,^[^ ^33^ ^]^ for precise A‐to‐G, C‐to‐T, and simultaneous A‐to‐G and C‐to‐T editing in rice, respectively (Figure 1A).

Currently, the most advanced plant dual‐base editors can be divided into two groups according to the required type of deaminase: 1) required both cytosine and adenine deaminases, with notable systems including STEME,^[^ ^14^ ^]^ PhieDBE,^[^ ^20^ ^]^ SWISS,^[^ ^15^ ^]^ and MoBE;^[^ ^16^ ^]^ 2) required single adenine deaminase variant, with notable systems including TadDE^[^ ^13^ ^]^ and rBE114a.^[^ ^35^ ^]^ These studies provided powerful tools to induce diverse nucleotide substitutions for plant genome editing. However, the STEME mostly induce C‐to‐T conversion rather than simultaneous C‐to‐T and A‐to‐G conversions, and the fusion position and order of deaminases significantly affect the editing efficiencies of STEME and PhieDBE systems.^[^ ^14^, ^20^ ^]^ Complicated constructions of the SWISS and MoBE systems raise technical threshold to achieve the desired dual‐base editing, as they require the expression of multiple protein effectors.^[^ ^15^, ^16^ ^]^ Recently, Fan et al.^[^ ^13^ ^]^ and Yu et al.^[^ ^35^ ^]^ fused single evolved TadA‐dual variant with PAM‐flexible Cas9 nickase, generating TadDE and rBE114a respectively, to enable simultaneous dual base editing at 5′‐NGN PAMs within a narrow editing window (≈5 nt, M_4_–M_8_) in rice. They provided streamlined construction and similar editing activities for both C‐to‐T and A‐to‐G editing. Whereas TadDE and rBE114a exhibit 5‐nt active window, TadDBE‐SpRYn exhibits further condensed editing window (≈3 nt, M_4_–M_6_) (Figure 1C). The highly condensed editing window allows specific single nucleotide or amino acid conversions, effectively mitigating bystander edits of adjacent nontarget nucleotides and avoiding frameshift mutations caused by InDel‐mediated errors (Figure 3B; Figure S9, Supporting Information). Moreover, introducing nearly PAM‐free SpRY nickase alleviates the constraints caused by the condensed editing window (3 nt) of the TadDBE‐mediated dual‐base editing for directed evolution, such as the gradient‐tuning enzyme activity of OsBadh2 in this case.

We also noticed that while TadDBE improved editing precision, it sacrificed some efficiency, which reflects the trade‐off between editing efficiency and precision. The 35S‐CmYLCV‐U6 composite promoter and tRNA system were successfully used to promote CRISPR/Cas9‐mediated genome editing,^[^ ^51^ ^]^ they may capable to enhance the editing efficiencies of TadDBE without compromising precision. Xiong et al.^[^ ^52^ ^]^ proposed a general strategy to minimize off‐target issues of BEs by splitting and complementation of deaminase and Cas9 nickase, which can reduce PAM‐flexible Cas9 variant‐induced off‐target effects. In addition, TadA‐dual can be fused with Transcription‐Activator‐Like Effector (TALE) and FokI nickase to develop single‐stranded DNA substrate for deamination,^[^ ^53^ ^]^ which may enable dual‐base editing in mitochondrial and chloroplast genomes. These features suggest that TadDBE‐SpRYn is particularly suitable for applications requiring precise editing of a single amino acid residue or targeting AT‐rich genomic regions.

Plant phenotypes can be fine‐tuned through methods such as promoter editing for gene expression regulation,^[^ ^54^ ^]^ uORF editing for translation inhibition,^[^ ^36^ ^]^ and point mutagenesis for protein engineering,^[^ ^14^, ^45^, ^46^ ^]^ which are essential approaches for breeding crops with desired traits. Our findings demonstrated that point mutagenesis provides a simple, universal, and predictable method for gradient‐tuning protein functions. Key functional residues of OsBadh2 were categorized into three groups based on their functional importance: 1) primary catalytic residues, 2) secondary substrate‐binding pocket residues, and 3) tertiary dimer‐linking residues (Figure 3A). Using TadDBE‐mediated dual‐base conversions, we generated diverse amino acid changes at 1–2 key functional residues (Figure 3B; Figure S9, Supporting Information). Unlike tiling mutagenesis, point mutagenesis targeting specific residues more efficiently modulated substrate‐enzyme binding efficiency or protein conformation, producing functionally gradient‐tuned variants (Figure 4A,B). Identification of critical residues, as well as precise point mutation‐mediated directed evolution, are essential. Recently, Wang et al.^[^ ^55^ ^]^ utilized AlphaFold‐guided bespoke *GmSWEET10b* editing to generate excellent artificial variations for enhancing soybean oil contents. This highlights the potential of deep learning‐based protein rational design and precise genome editing for crop genetic improvement. This approach can be extended to regulate quantitative agronomic traits such as plant height, heading stage, and amylose content.

Balancing tradeoffs caused by gene pleiotrophy is crucial for improving crop traits. Both 2‐AP and GABA are derived from the same upstream substrate, GABald, with OsBadh2 acting as a molecular switch in this pathway. The complete loss‐of‐function of *OsBadh2*, common in fragrant rice varieties, results in low GABA levels, whereas nonfragrant rice lacks aroma, reducing taste quality (Table 1; Figure S10E, Supporting Information). By precisely designing endogenous proteins and gradient‐tuning *OsBadh2* enzyme activity, we developed rice germplasms with moderate and balanced 2‐AP and GABA contents (Figure 4A,B). These findings underscore the potential of biofortification breeding to harmonize nutritional quality and flavor.

Finally, our data revealed a strong correlation between ΔΔ*G* variations and protein activity in mutant plants (Figure 4C). This suggests that artificial intelligence‐based deep training models could reliably simulate advanced protein structures and predict molecular docking results, offering a predictive framework for molecular experimentation.

In conclusion, we characterized the base editing performances of three TadA‐derived BEs with condensed editing window and PAM‐flexible targeting scopes for precise heterogeneous nucleotide conversions in rice. Through dual‐base editing‐mediated point mutagenesis, we generated gradient‐tuned rice aroma and GABA content, showcasing the feasibility of precise genome editing and universal gradient tuning of protein function tuning. These TadA variants and future optimizations will significantly advance plant research and accelerate crop breeding.

## 4 Experimental Section

### Plant Materials and Growing Conditions

The *japonica* cultivar Nipponbare (NIP) was chosen as the background genetic materials for genome editing at target sites (TS) TS1–TS13 in calli and site‐saturation mutagenesis of *OsBadh2* in plants. The wild‐type and edited NIP lines, along with a fragrant *japonica* cultivar DaoHuaXiang (DHX) were grown in paddy fields in Guangzhou (113°43′E, 23°39′N) under natural growing conditions, in preparation for metabolite determination. Several key agronomic traits, including plant height, tiller number, number of primary branches, number of secondary branches, number of spikelets per panicle, and 1000‐grain weight of wild type and mutants were analyzed after ripening.

### Vector Construction

The rice codon‐optimized TadA9 (V106W, N108Q, L145T), TadA‐LM (N46L, A48M, V106W), TadA‐dual (R26G, V28A, A48R, Y73S, H96N, V106W) variants and SpRY nickase (SpRYn) were generated from PhieABEs^[^ ^21^ ^]^ using PCR. The hyDBE‐SpRYn was constructed by replacing SpGn with SpRYn in PhieDBE.^[^ ^20^ ^]^ All the base editors (BEs) were attached to bpNLS on both sides, and cloned into the pYLCRISPR/Cas9Pubi‐H vector^[^ ^56^ ^]^ using the modified Gibson cloning method.^[^ ^57^ ^]^ All primers used in this study are listed in Table S6 (Supporting Information).

### Target Design and Assembly of sgRNA Expression Cassette

To systematically evaluate the editing characteristic of various precise BEs, 13 target sites (TS1–TS13) with 5′‐NNN PAM were selected from 12 endogenous rice genes (Table S1, Supporting Information) by using the online webtool CRISPR‐GE (http://skl.scau.edu.cn/).^[^ ^58^ ^]^ Sites TS14–TS27 located at different functional residues of OsBadh2 were designed based on relative position between the editing window of TadDBE and the target nucleotides in *OsBadh2*, facilitating site‐saturation mutagenesis‐mediated gradient‐tuned protein design. A pYLCRISPR/Cas9Pubi‐H construct with single sgRNA cassette (Table S6, Supporting Information) was utilized to generate *badh2* knockout mutants. Predicted homologous off‐target loci with ≤ 2‐nucleotide mismatches were identified using CRISPR‐GE. The assembly of expression cassettes containing multiple sgRNAs for multiplex editing was performed using Golden‐Gate cloning. All combinations of sgRNA expression cassettes assembled into BEs are detailed in Tables S1 and S4 (Supporting Information).

### Agrobacterium Tumefaciens‐Mediated Rice Transformation

All constructs were transformed into the NIP via *Agrobacterium tumefaciens*‐mediated transformation using strain EHA105. The rice transformation service was provided by Wuhan Edgene biotechnology Co., Ltd and Wuhan Boyuan biotechnology Co., Ltd. Transgenic calli or plants (T_0_) PCR‐positive for the hygromycin phosphotransferase gene *HPT* and *Cas9* were used for further analyses.

### NGS Library Preparation and Mutagenesis Analysis

Rice genomic DNA was isolated from all T_0_ calli or plants via the cetyltrimethylammonium bromide method. The genomic regions flanking sgRNA target sites, sgRNA cassettes within the T‐DNA region, and the predicted off‐target loci were amplified using specific primers listed in Table S6. Several mixed libraries were prepared for mutagenesis analysis according to the Hi‐TOM's protocol.^[^ ^59^ ^]^ The resulting PCR products (≈220–230 bp) were sequenced through next‐generation sequencing (NGS) on an Illumina HiSeq platform. Mutation types and base conversion frequencies were calculated from at least 1.0 Gb of data per sample, which included 96 × 1 to 96 × 4 genome‐modified sites. The sequencing results were aligned to the reference genome and analyzed via Hi‐TOM platform with a filter threshold of 15%. Low‐frequency edits below this threshold were excluded from the analysis. To test the accuracy of NGS, samples with low reads were validated by comparing them with the results of Sanger sequencing. To distinguish background mutations, the positions with ≤ 2 mismatches to the target sites were amplified, and mutations generated at these potential off‐target sites were considered to be bona fide off‐target edits.

### Simulation of Protein Structure

The protein structures of TadA variants and OsBadh2 mutants were predicted using Alphafold 3.^[^ ^47^ ^]^ All figures of structural models were developed using PyMOL (Version 2.6.0a0).

### Molecular Docking

The AlphaFold‐predicted structures of OsBadh2 and derived mutants were downloaded from https://alphafoldserver.com/. The structure of GABald was downloaded from https://pubchem.ncbi.nlm.nih.gov/. Molecular docking simulations were performed using the commercial software AMDock (Version 1.5.2).^[^ ^60^ ^]^ The binding capacity of GABald to the novel OsBadh2 proteins was predicted based on the binding affinity (Δ*G*), which is a score of the free energy of the protein bound to substrate and calculated as Δ*G* = *RT*ln(*K*m). The binding affinity change upon mutation is defined as ΔΔ*G* = Δ*G*_mutant_ – Δ*G*_WT_. Positive and negative values of ΔΔ*G* correspond to the mutations decreasing and increasing binding affinity, respectively.

### Determination of 2‐AP and GABA Content

For each tested lines, three or more biological replicates were required. The rice grains were dehulled and subsequently grounded into a fine powder using a high‐speed grinder. A mass of 1.00 g rice powder was accurately weighed and mixed with 1.5 mL absolute alcohol as extraction buffer. The mixture was placed in a sealed container and subjected to an extraction process at 68 °C for 2 h in ultrasound bath. Following the extraction, the mixture was centrifuged at 10,000 × *g*, 4 °C for 10 min to separate the solids from the liquid phase. The resulting clear supernatant was then filtered through a 0.22 µm pore‐size organic filter membrane to ensure the removal of any particulate matter, and prepared for examination.

The quantification of 2‐acetyl‐1‐pyrroline (2‐AP) levels in various rice lines was performed using gas chromatography‐mass spectrometry (GC‐MS). The Agilent 7890 A GC system, coupled with the Agilent 7000 D triple quadrupole mass detector was used for the detection of 2‐AP. Helium gas of 99.999% purity served as the carrier, with the injector and GC‐MS interface temperatures set at 250 °C. The separation was achieved on a HP‐5 MS capillary column (30 m × 0.25 mm × 0.25 µm), which was initially maintained at 45 °C, then temperature was ramped up to 250 °C at a rate of 8 °C∙min^−1^, after holding for 1 min at the initial temperature. The eluent was introduced directly into the mass spectrometer, operated in electron impact mode with an ionization voltage of 70 eV and an ion source temperature of 250 °C. The reference retention time for 2‐AP is 6.50 min, with *m*/*z* 111.0‐83.1 as quantitative ion pair. The 2‐AP content was quantified using the following equation: *ω* = (*ρ* – *ρ_0_*) × *V* × *m* ^−1^. *ω*: 2‐AP content (µg∙kg^−1^); *ρ*: sample concentration (µg∙mL^−1^); *ρ_0_*: blank concentration (µg∙mL^−1^); *V*: sample size (µL); *m*: sample weight (g).

For standard quantification, the 1 mg∙mL^−1^ 2‐AP mother solution was prepared by dissolving 2‐AP (with a purity ≥ 95%, CAS NO: 85213‐22‐5) in absolute alcohol. Subsequently, the standard solutions with concentrations of 0.05, 0.1, 0.25, 0.5, 1.0 and 2.5 µg∙mL^−1^ were prepared. A standard calibration curve was then constructed based on the 2‐AP peak area and the corresponding injected 2‐AP content.

High‐performance liquid chromatography (HPLC) analysis was performed for measurement of γ‐aminobutyric acid (GABA) levels by using an Agilent 1260 LC analyzer. Solution composed of A (75% acetonitrile) and B (0.1 mol∙L^−1^ NaAc) served as the carrier, with the Information‐HILICZ column (2.7 µm, 100 mm × 3.0 mm) temperatures set at 35 °C. The detected conditions were as follows: 10% A at 0–5 min; a linear gradient to 50% A at 5–8 min; a linear gradient to 10% A held to the end of analysis (15 min). The flow rate was fixed at 0.3 mL∙min^−1^ with a detection wavelength at 436 nm. The GABA content was quantified using the following equation: *ω* = *ρ* × *V* × *m* ^−1^. *ω*: GABA content (mg∙kg^−1^); *ρ*: sample concentration (µg∙L^−1^); *V*: sample size (mL); *m*: sample weight (g). The GABA standard (with a purity ≥ 99.9%, CAS NO: 56‐12‐2) was diluted into a gradient solution (1.0, 5.0, 25.0, 50.0, 250.0 and 500.0 µg∙L^−1^) and used to generate standard curves for quantitative analysis of the rice grain samples.

### Functional Modeling and Statistical Analysis

Functional modeling was generated using Microsoft Office Excel based on scatter plot. Graphic representations in this study were generated using GraphPad Prism 8 or Microsoft Office PowerPoint. Variance analysis and significance testing (*P* < 0.05) were conducted using SPSS 18.0, where mean values and standard errors (SE) were calculated. For the purpose of multiple comparisons, the Duncan's shortest significant ranges (SSR) method was applied.

## Conflict of Interest

The authors declare no conflict of interest.

## Author Contributions

Conceptualization, J. T.; Methodology, K. S., S. C., N. C., J. M., R, Z., Q. Q., and Z. Z.; Investigation, K. S., K. C., D. Z., X. P., M. S. and D. Z.; Writing – Original Draft, K. S. and J. T.; Writing – Review & Editing, Q. Z. and J. T.; Funding Acquisition, Q. Z., Q. L. and J. T.; Resources, Q. Z. and Q. L.; Supervision, J. T.

## Supporting information



Supporting Information

## Data Availability

The data that support the findings of this study are available in the supplementary material of this article.;
