# Supplementary material for: Engineering Adenine Deaminase TadA for Precise and PAM‐Flexible Point Mutagenesis and Gradient‐Tuning Endogenous Protein Design
Source: Adv Sci (Weinh). 2025 Jun 20;12(34):e06644. doi: 10.1002/advs.202506644 (PMC12442613; doi:10.1002/advs.202506644)
Supplement: Supplementary file 1 — Supporting Information [file ADVS-12-e06644-s001.docx]

**Supporting Information**

**Figure S1.** Nucleotide sequences of TadA9, TadA-LM and TadA-dual variants, and functional domains of OsBadh2.

**Figure S2.** Rendering of the crystal structures of TadA variants.

**Figure S3.** Preference analyses of ABE9, CBEm, TadDBE and hyDBE at the target A and C in different sequence contexts.

**Figure S4.** Self-target analysis of base editors in the sgRNA expression cassettes at TS1–TS13 sites.

**Figure S5.** Off-target analysis of base editors at *IPI1*-TS2-, *TubA2*-TS3-, *TT3.2-*TS6-, *NRT1.1B*-TS7-, *TT3.1*-TS9-, *bsr-d1*-TS11-, *PAY1*-TS12- and *CPK4*-TS13-homologous sites in this study.

**Figure S6.** DBEs broaden the range of achievable amino acid conversions.

**Figure S7.** Structural characterization of mutation residues in the atomic models of novel OsBadh2 variants.

**Figure S8.** Dimeric OsBadh2 with candidate key residues at the interface.

**Figure S9.** Amino acid substitutions introduced by TadDBE at the key functional sites of OsBadh2.

**Figure S10.** Gas Chromatograph-Mass Spectrometry (GC-MS) analysis of 2-AP and high-performance liquid chromatography (HPLC) analysis of GABA in rice grain.

**Figure S11.** Statistics of the key agronomic traits in NIP, *badh2*, DHX and TadDBE-edited plants.

**Table S1.** Base editing efficiencies of ABE9, CBEm, TadDBE and hyDBE at 13 endogenous targets with 5′-NNN PAMs in rice calli.

**Table S2.** The number of analyzed calli and mutated calli with multiplex genome editing showed in Figure 2C.

**Table S3.** The number of analyzed calli and mutation types at each target site showed in Figure 2D.

**Table S4.** Nucleotide substitutions, amino acid changes and editing frequencies induced by TadDBE at *OsBadh2* in T_0_ plants.

**Table S5.** TadA-derived editors compared to other recently reported base editors.

**Table S6.** Oligos used in this study.

**
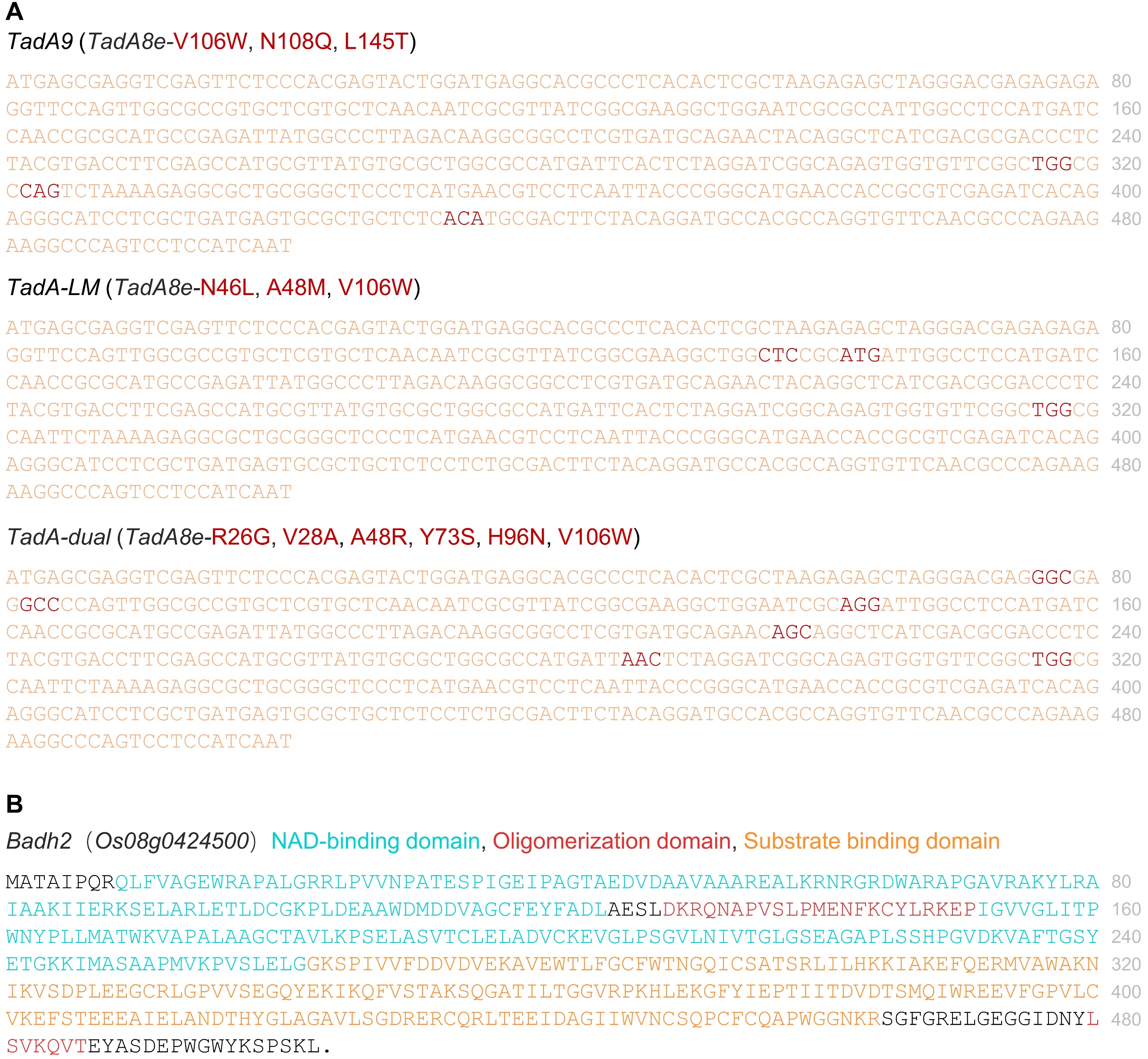
**

**Figure S1.** Nucleotide sequences of TadA9, TadA-LM and TadA-dual variants, and functional domains of OsBadh2.

**(A)** Nucleotide sequences of rice codon-optimized TadA9, TadA-LM and TadA-dual variants. The triplet codons encoding mutated amino acids are highlighted in crimson. **(B)** Protein sequence of OsBadh2. NAD-binding domain (blue), oligomerization domain (red), and substrate binding domain (orange) are showed.

**
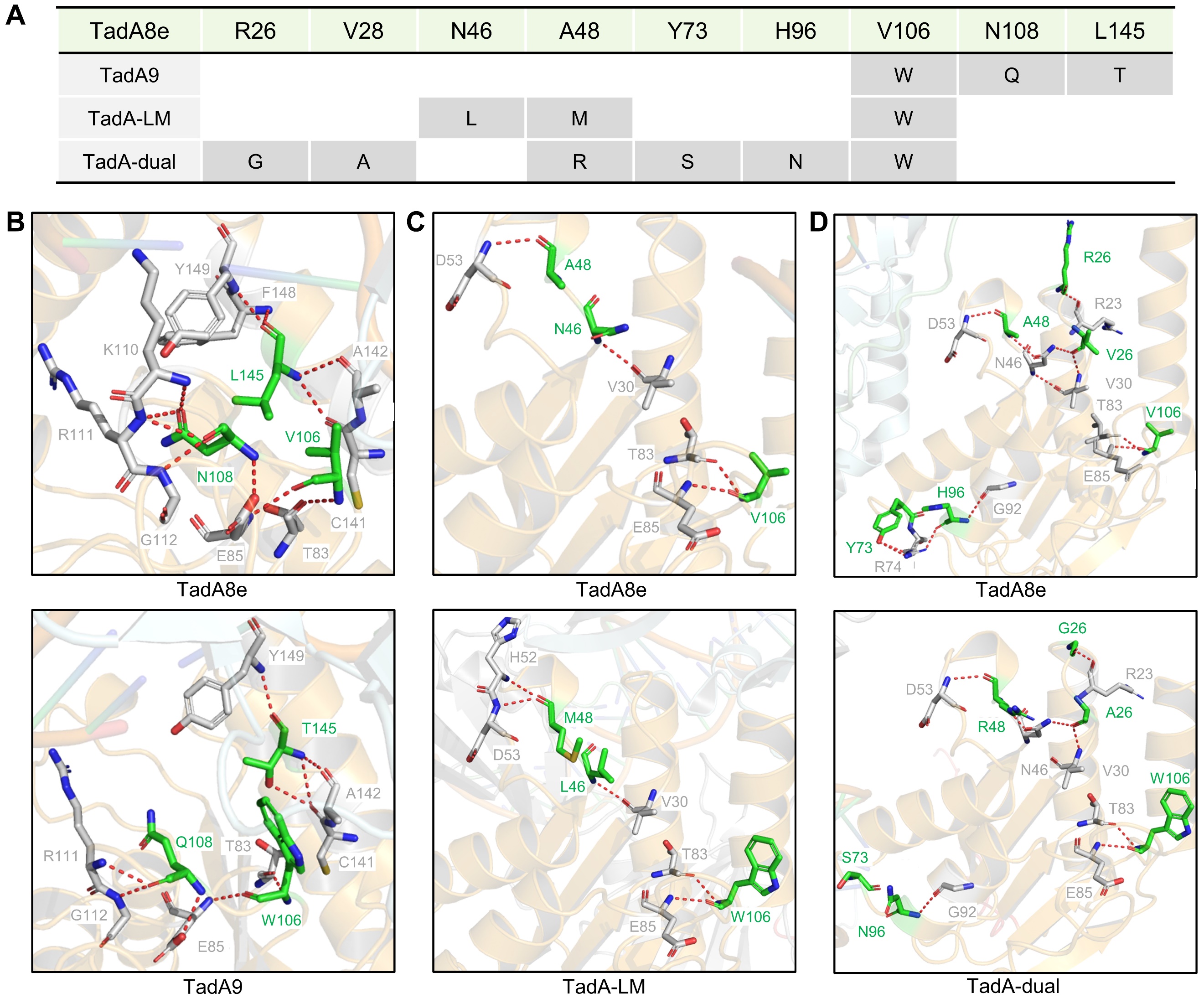
**

**Figure S2.** Rendering of the crystal structures of TadA variants.

**(A)** Summary of the variabilities among TadA variants. **(B–D)** Structural modeling of TadA9 **(B)**, TadA-LM **(C)** and TadA-dual **(D)** compared with TadA8e. The mutant and interacting residues are showed in green and grey, respectively. Images were generated using AlphaFold 3.

**
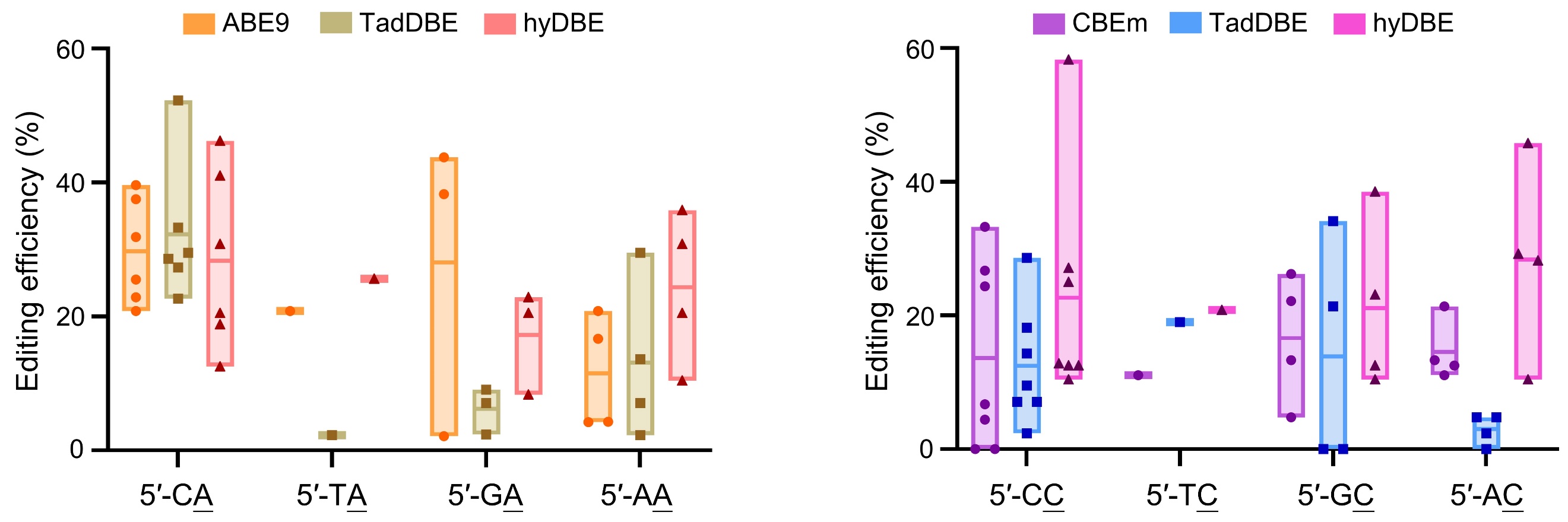
**

**Figure S3.** Preference analyses of ABE9, CBEm, TadDBE and hyDBE at the target A and C in different sequence contexts.

The editing site is underlined.

**
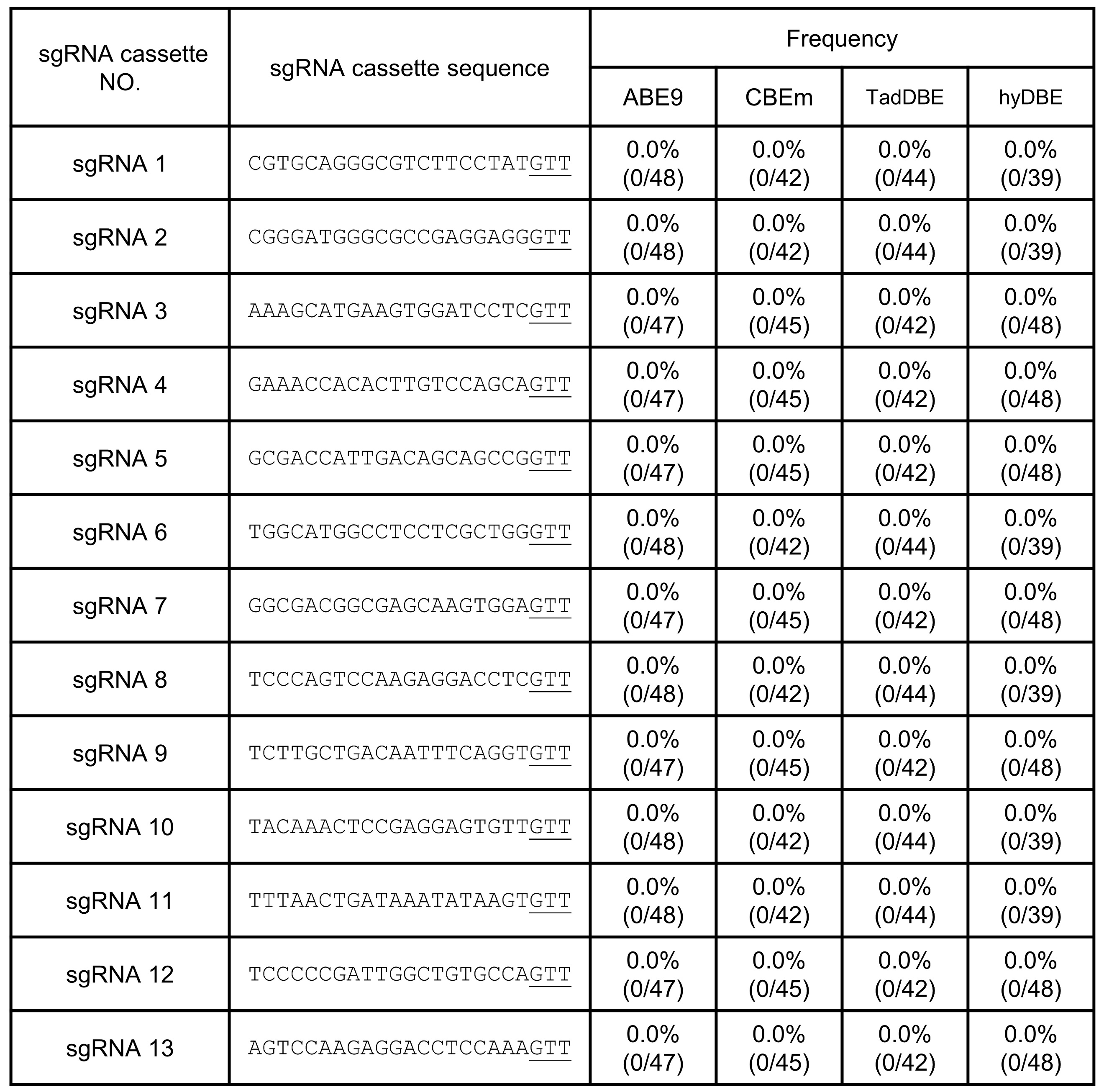
**

**Figure S4.** Self-target analysis of base editors in the sgRNA expression cassettes at TS1–TS13 sites.

PAM sequences (5′-GTT) in sgRNA cassettes are underlined. The self-target editing frequencies of each sgRNA cassette are showed as the proportion of mutants, harboring any type of mutation at sgRNA cassette sequences. The values are calculated by number of mutants divided by total number of transgenic calli.

**
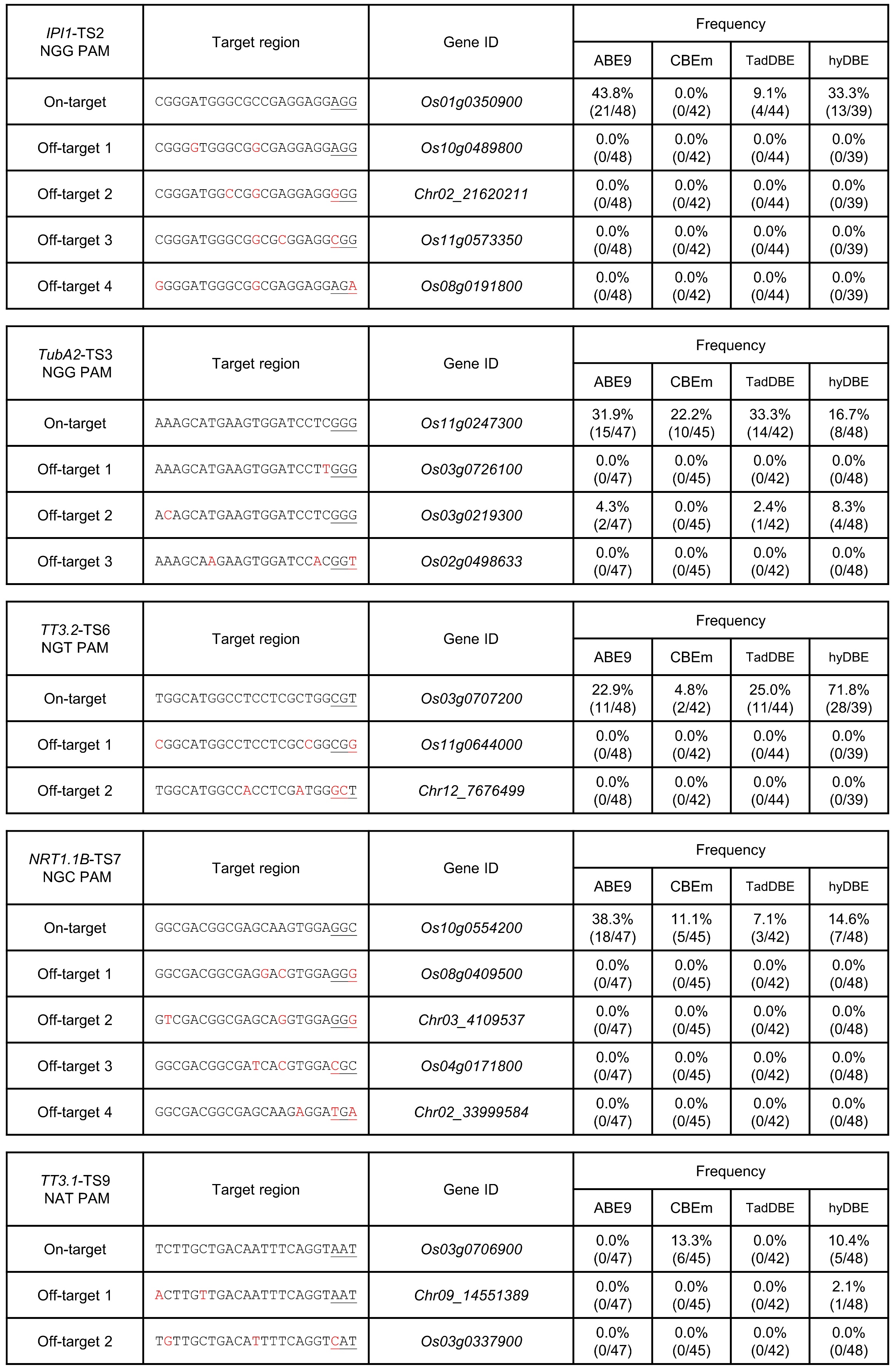

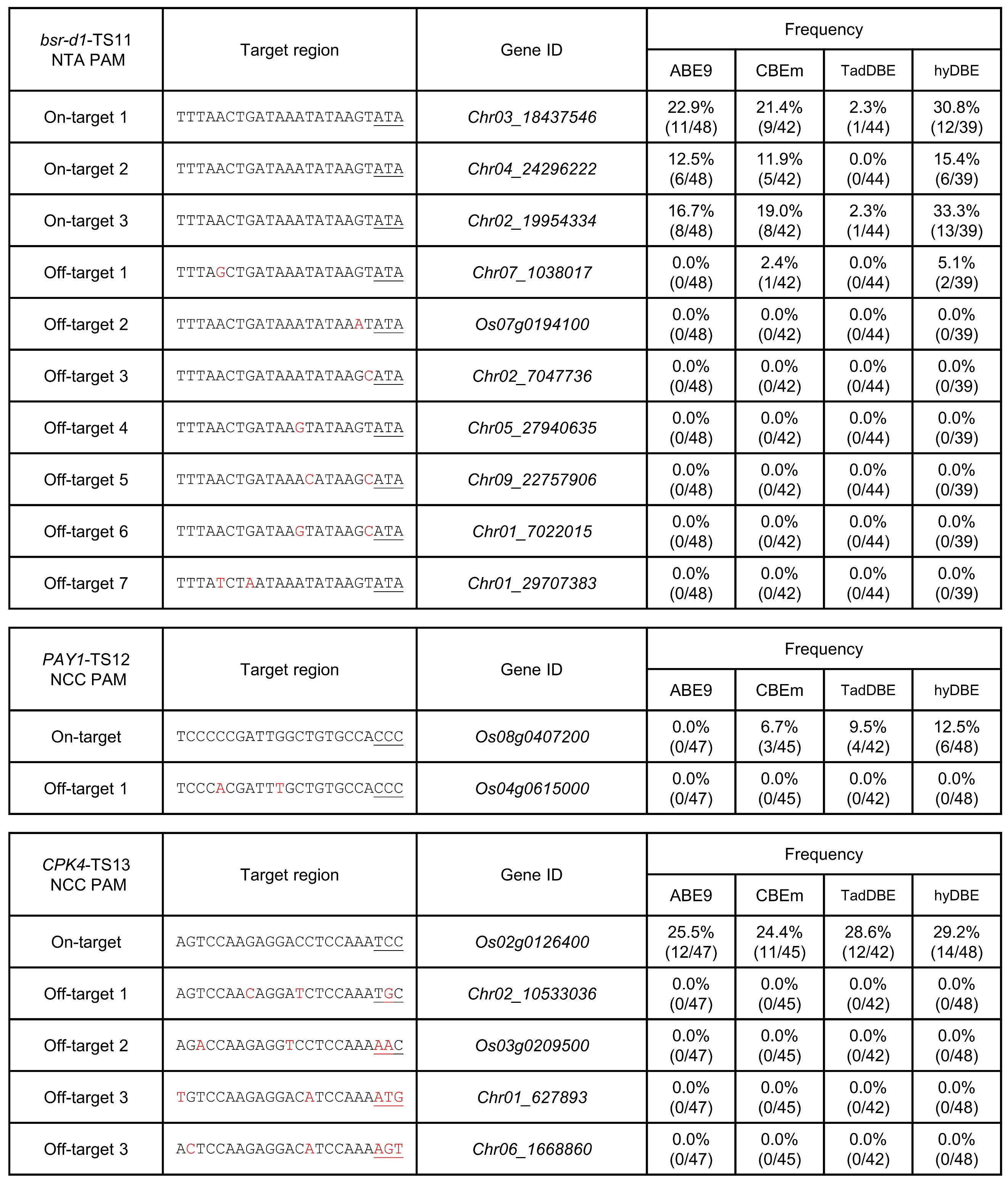
**

**Figure S5.** Off-target analysis of base editors at *IPI1*-TS2-, *TubA2*-TS3-, *TT3.2-*TS6-, *NRT1.1B*-TS7-, *TT3.1*-TS9-, *bsr-d1*-TS11-, *PAY1*-TS12- and *CPK4*-TS13-homologous sites in this study.

PAM sequences are underlined; mismatches in the sgRNA sequences are highlighted in red. The on-target and off-target editing frequencies of each tested locus are showed as the proportion of mutants, harboring any type of mutation at the target. The values are calculated by number of mutants divided by total number of transgenic calli.

**
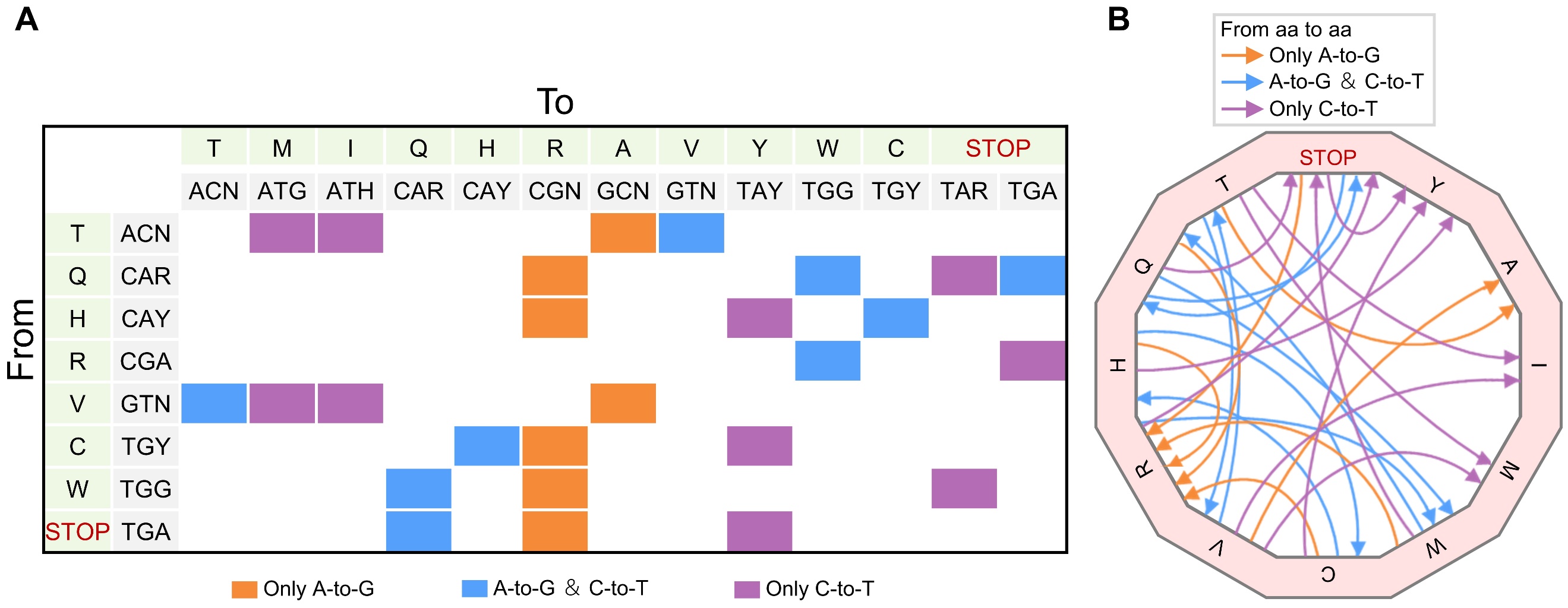
**

**Figure S6.** DBEs broaden the range of achievable amino acid conversions.

**(A)** Particular amino acid changes at codons edited by DBEs. R = A or G; Y = C or T; H = A, C or T; N = A, C, T or G. **(B)** Circos plot illustrates the types of amino acid substitutions induced by DBEs, including amino acid changes resulting from single A-to-G conversions (orange), single C-to-T conversions (purple) and dual-base conversions which are uniquely enabled (blue).


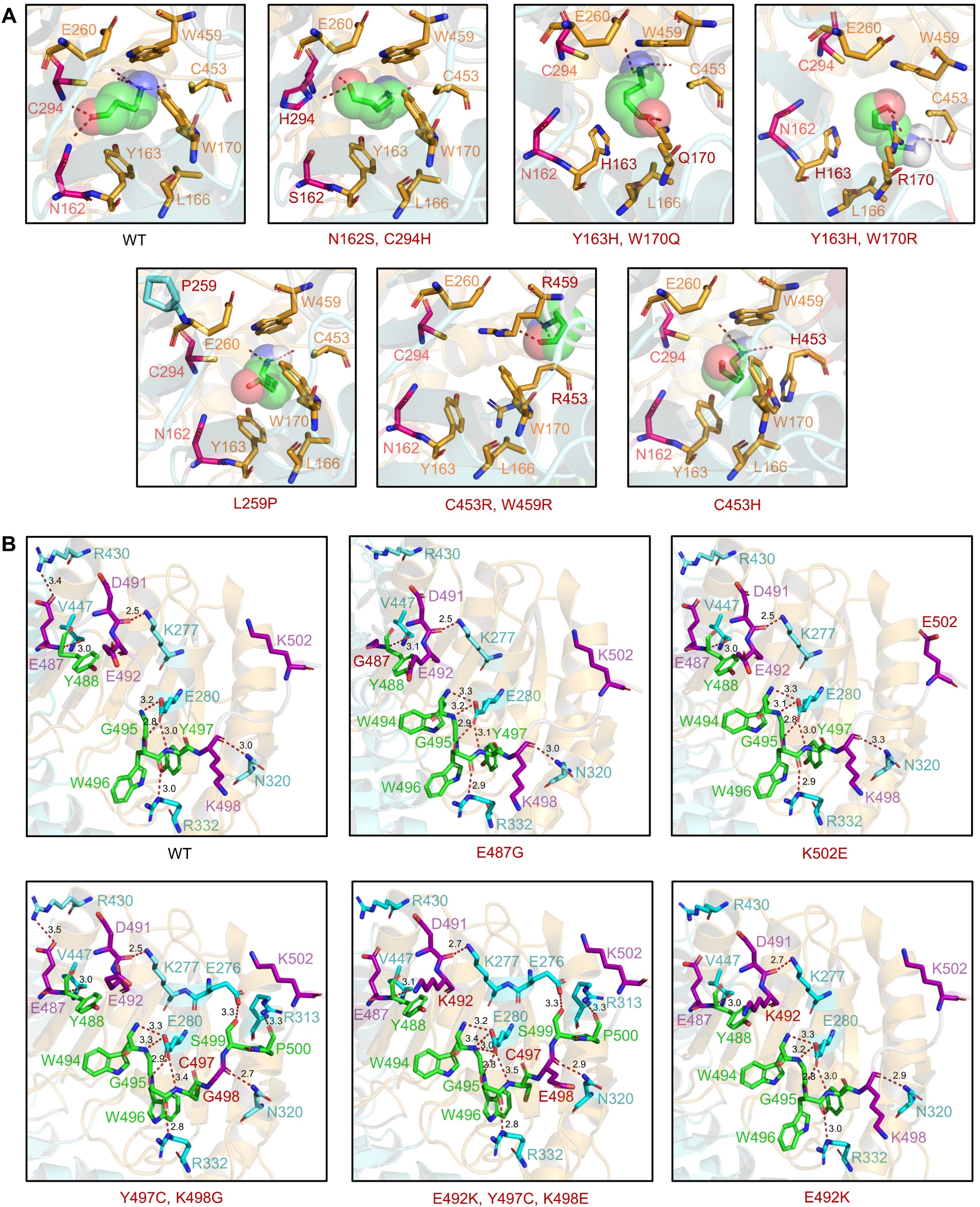


**Figure S7.** Structural characterization of mutation residues in the atomic models of novel OsBadh2 variants.

**(A)** Structural modeling of the interaction between GABald and the catalytic pockets of various OsBadh2 mutants, as predicted by AMDock. The predicted hydrogen bond connections between GABald and the residues are marked by red dashed lines. **(B)** The generation and extinction of hydrogen bonds, along with changes in distances among key residues at the dimer interface of OsBadh2 mutants are displayed. Five key charged residues are highlighted in purple. Other residues located on different subunits are marked in green and blue, respectively. Mutated residues are marked in crimson.

**
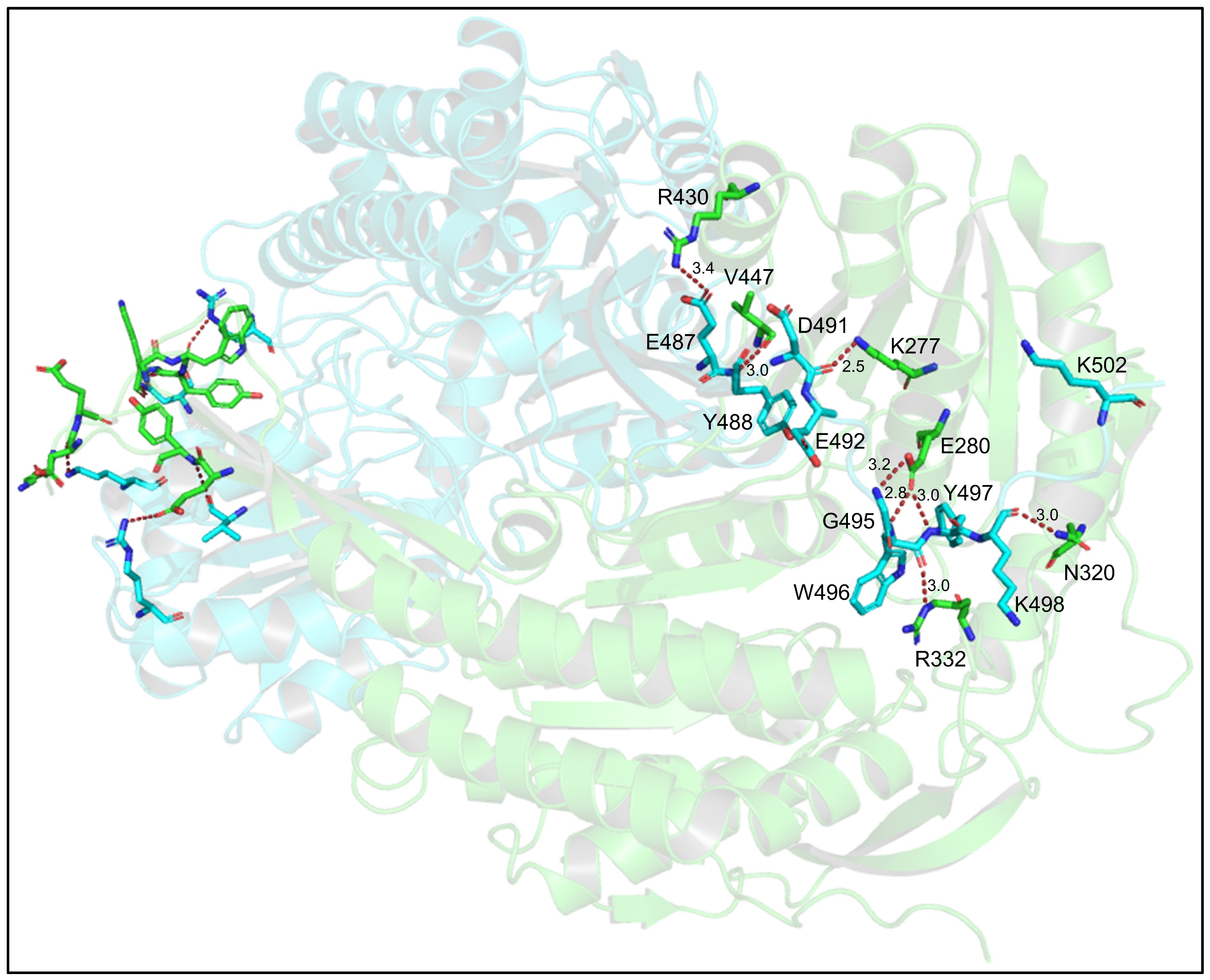
**

**Figure S8.** Dimeric OsBadh2 with candidate key residues at the interface.

The interacting residues distributed across different subunits are marked in green and blue, respectively. The predicted hydrogen bond connections are indicated in red, along with atomic distances. Image was generated using AlphaFold 3.

**
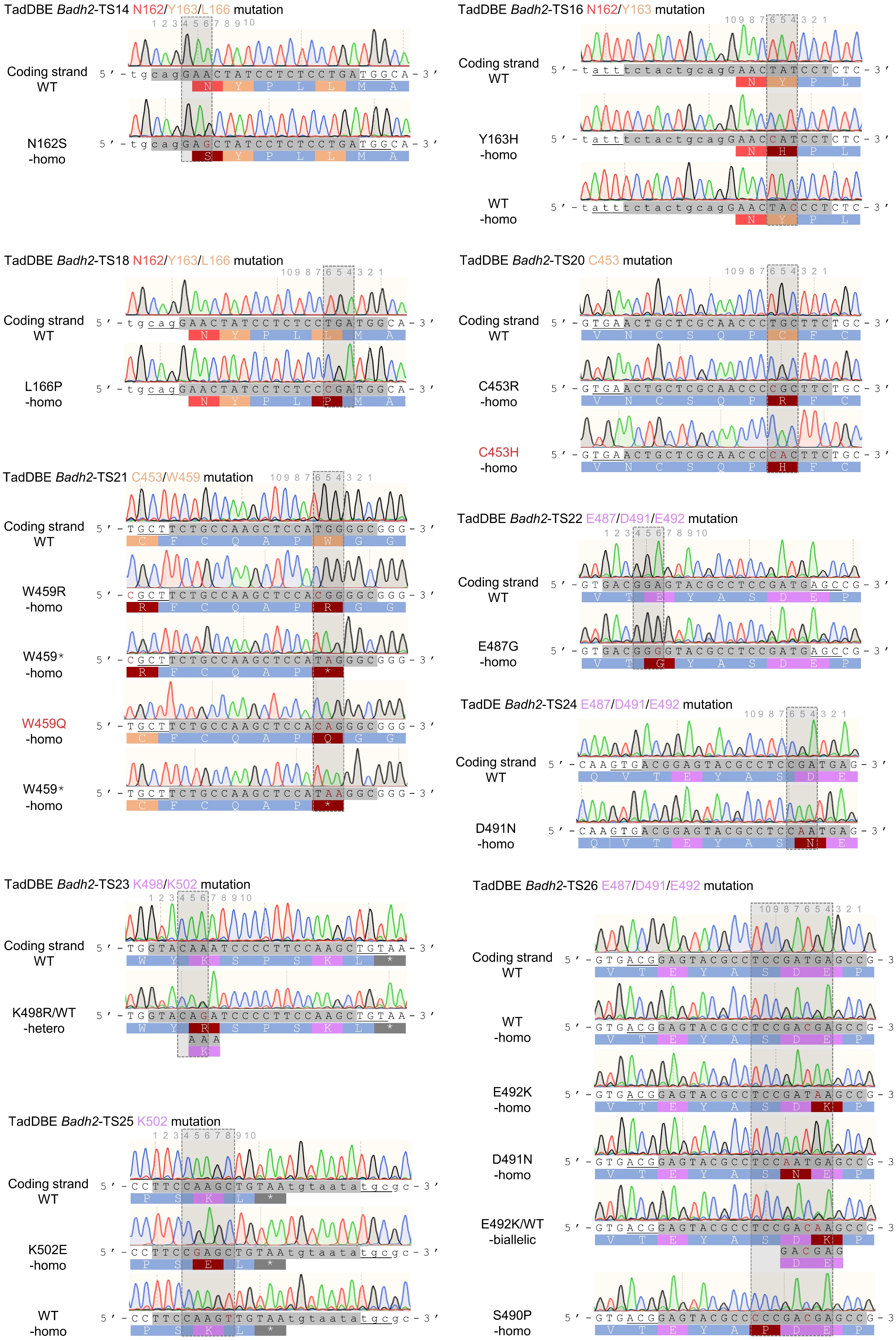
**

**Figure S9.** Amino acid substitutions introduced by TadDBE at key functional sites of OsBadh2.

The mutated nucleotides and amino acids are highlighted in crimson and crimson background, respectively.

**
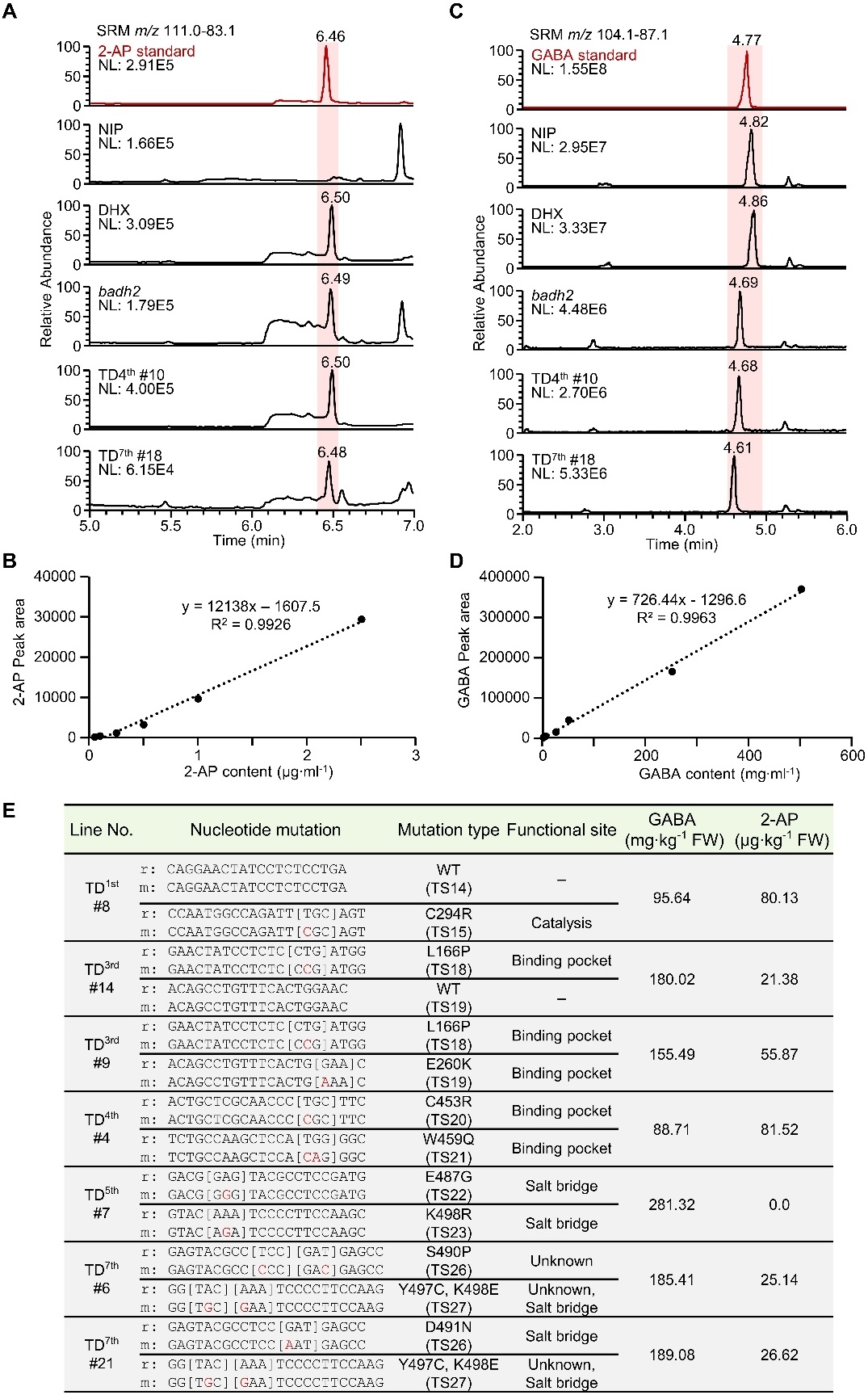
**

**Figure S10.** Gas Chromatograph-Mass Spectrometry (GC-MS) analysis of 2-AP and high-performance liquid chromatography (HPLC) analysis of GABA in rice grain.

**(A)** Peaks corresponding to 2-AP in standard of 2-AP, Nipponbare (NIP), DaoHuaXiang (DHX), *badh2* (knock-out mutant), TD4^th^ #10 and TD7^th^ #18 mutants were identified and analyzed using GC-MS. **(B)** Calibration curve of 2-AP. **(C)** Peaks corresponding to GABA in standard of GABA, NIP, DHX and mutants were identified and analyzed using HPLC. **(D)** Calibration curve of GABA. **(E)** 2-AP and GABA contents in rice grains of several mutants that supplement Table 1.

**
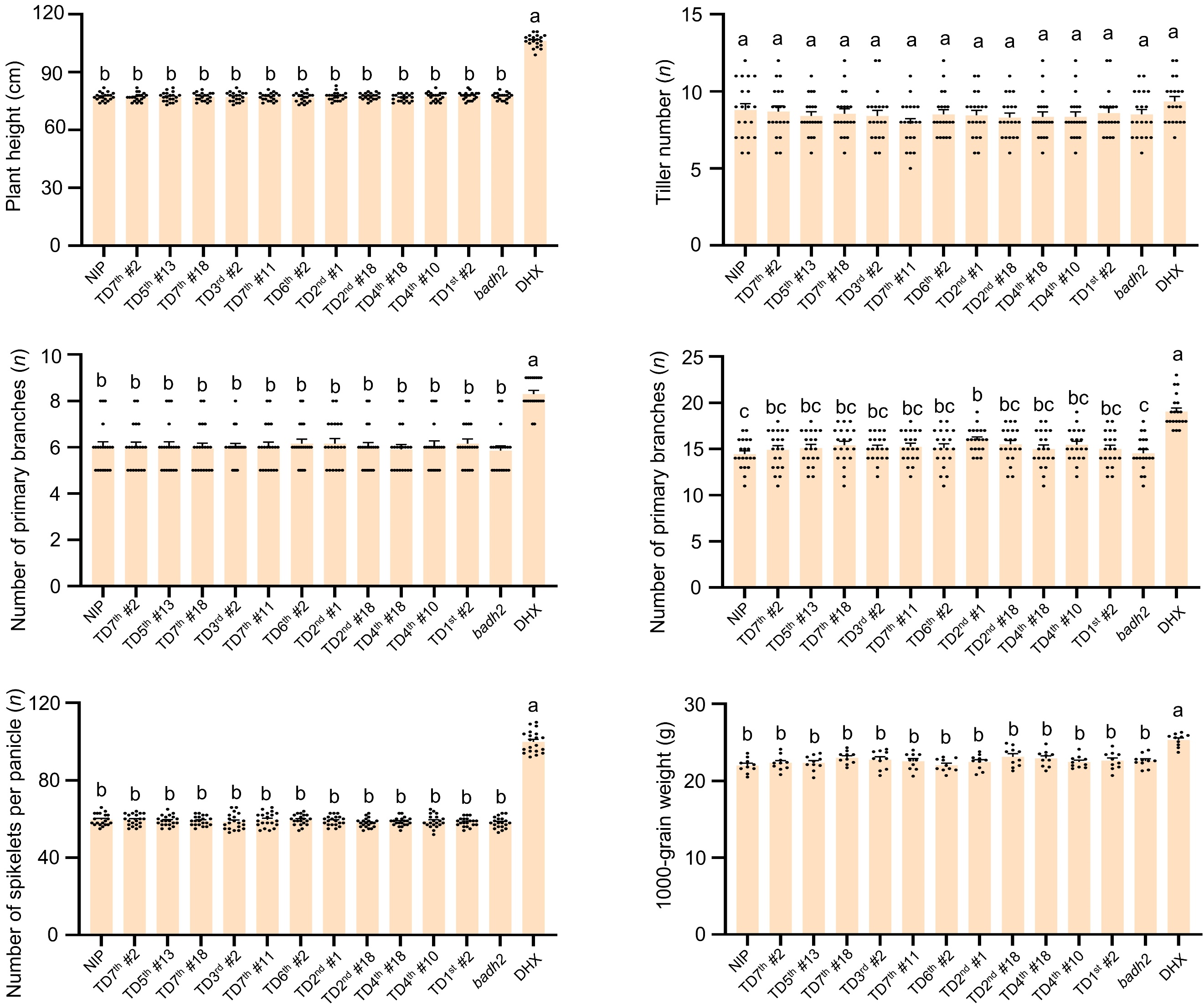
**

**Figure S11.** Statistics of the key agronomic traits in NIP, *badh2*, DHX and TadDBE-edited plants.

Plant height, tiller number, number of primary branches, number of secondary branches, number of spikelets per panicle, and 1000-grain weight of NIP, *badh2*, DHX and several TadDBE-edited plants were analyzed. Means with the same letter are not significantly different, as assessed by Duncan's multiple range test (*P* < 0.05). Data are mean ± SE (*n* = 20 for plant height, tiller number, number of primary branches, number of secondary branches, and number of spikelets per panicle; *n* = 10 for 1000-grain weight).

**Table S1.** Base editing efficiencies of ABE9, CBEm, TadDBE and hyDBE at 13 endogenous targets with 5′-NNN PAMs in rice calli.

| Target NO. | Target sequence (5′–3′) | ABE9 | CBEm | | TadDBE | | hyDBE | |  |
| --- | --- | --- | --- | --- | --- | --- | --- | --- | --- |
| *bsr-k1-*TS1 | CGTGCAGGGCGTCTTCCTATGGG | 39.6%  (19/48) | 26.2%  (11/42) | | 52.3%  (23/44) | | 46.2%  (18/39) | |  |
| *IPI1-*TS2 | CGGGATGGGCGCCGAGGAGGAGG | 43.8%  (21/48) | 0%  (0/42) | | 9.1%  (4/44) | | 33.3%  (13/39) | |  |
| *TubA2-*TS3 | AAAGCATGAAGTGGATCCTCGGG | 31.9%  (15/47) | 22.2%  (10/45) | | 33.3%  (14/42) | | 16.7%  (8/48) | |  |
| *ACCase-*TS4 | GAAACCACACTTGTCCAGCACGA | 4.3%  (2/47) | 31.1%  (14/45) | | 14.3%  (6/42) | | 75.0%  (36/48) | |  |
| *EPSPS-*TS5 | GCGACCATTGACAGCAGCCGTGA | 2.1%  (1/47) | 37.8%  (17/45) | | 7.1%  (3/42) | | 41.7%  (20/48) | |  |
| *TT3.2-*TS6 | TGGCATGGCCTCCTCGCTGGCGT | 22.9%  (11/48) | 4.8%  (2/42) | | 25.0%  (11/44) | | 71.8%  (28/39) | |  |
| *NRT1.1B-*TS7 | GGCGACGGCGAGCAAGTGGAGGC | 38.3%  (18/47) | 11.1%  (5/45) | | 7.1%  (3/42) | | 14.6%  (7/48) | |  |
| *CPK4-*TS8 | TCCCAGTCCAAGAGGACCTCCAA | 37.5%  (18/48) | 0%  (0/42) | | 29.5%  (13/44) | | 56.4%  (22/39) | |  |
| *TT3.1-*TS9 | TCTTGCTGACAATTTCAGGTAAT | 0%  (0/47) | 13.3%  (6/45) | | 0%  (0/42) | | 10.4%  (5/48) | |  |
| *CPK18-*TS10 | TACAAACTCCGAGGAGTGTTCAC | 58.3%  (28/48) | 0%  (0/42) | | 61.4%  (27/44) | | 46.2%  (18/39) | |  |
| *bsr-d1-*TS11 | TTTAACTGATAAATATAAGTATA | 22.9%  (11/48) | 21.4%  (9/42) | | 2.3%  (1/44) | | 30.8%  (12/39) | |  |
| *PAY1-*TS12 | TCCCCCGATTGGCTGTGCCACCC | 0%  (0/47) | 6.7%  (3/45) | | 9.5%  (4/42) | | 12.5%  (6/48) | |  |
| *CPK4-*TS13 | AGTCCAAGAGGACCTCCAAATCC | 25.5%  (12/47) | 24.4%  (11/45) | | 28.6%  (12/42) | | 29.2%  (14/48) | |  |
| Average editing efficiencies  at the 4^th^ nucleotide* | | 4.2% for A | | 0% for A | | 7.6% for A | | 2.3% for A | |
|  |  | 0% for C | | 1.1% for C | | 15.5% for C | | 11.5% for C | |
| Average editing efficiencies  at the 5^th^ nucleotide* | | 37.3% for A | | 0% for A | | 13.7% for A | | 36.6% for A | |
|  |  | 0% for C | | 24.0% for C | | 17.3% for C | | 32.3% for C | |
| Average editing efficiencies  at the 6^th^ nucleotide* | | 33.7% for A | | 0% for A | | 41.9% for A | | 31.0% for A | |
|  |  | 0% for C | | 19.5% for C | | 1.6% for C | | 25.4% for C | |
| Total editing efficiencies within the editing windows^ | | 25.3% for A | | 0% for A | | 20.0% for A | | 18.3% for A | |
|  |  | 0% for C | | 15.5% for C | | 11.0% for C | | 20.2% for C | |

Note: In target sequences, adenine (A) edited to guanine (G) are highlighted in orange. Cytosine (C) edited to D [thymine (T), G or A] are highlighted in purple. PAMs are highlighted in underlines. The proportion of mutants at each target site for each base editor are showed on the right of each target sequence, calculated by number of mutated calli (including homozygous, heterozygous, biallelic nucleotide substitutions and InDels) divided by total number of transgenic calli.

*, average editing efficiencies for A and C nucleotides at specific 4^th^, 5^th^, and 6^th^ positions. The values are calculated by using the following equation: A-to-G frequency = *N_1_* × *N**_2_*^-1^ and C-to-D frequency = *N_3_* × *N_4_*^-1^. *N_1_*: number of alleles with A-to-G edits at specific position (the 4^th^, 5^th^, or 6^th^ position); *N_2_*: number of alleles with A nucleotide at specific position (the 4^th^, 5^th^, or 6^th^ position); *N_3_*: number of alleles with C-to-D edits at specific position (the 4^th^, 5^th^, or 6^th^ position); *N_4_*: number of alleles with C nucleotide at specific position (the 4^th^, 5^th^, or 6^th^ position).

^, total editing efficiencies for A and C nucleotides within the editing windows. The values are calculated by using the following equation: A-to-G frequency = *N_5_* × *N_7_*^-1^ × 2^-1^ and C-to-D frequency = *N_6_* × *N_7_*^-1^ × 2^-1^. *N_5_*: number of alleles with A-to-G edits; *N_6_*: number of alleles with C-to-D edits; *N_7_*: total number of transgenic positive calli.

**Table S2.** The number of analyzed calli and mutated calli with multiplex genome editing showed in Figure 2C.

| Number of sgRNA cassettes | BEs | Total number of tested calli | Number of mutated calli | Number of mutated calli with multiplex genome editing | | | | | | |
| --- | --- | --- | --- | --- | --- | --- | --- | --- | --- | --- |
|  |  |  |  | 1 | 2 | 3 | 4 | 5 | 6 | 7 |
| 6 | ABE9 | 48 | 29 | 8  (16.7%) | 0  (0.0%) | 2  (4.2%) | 10  (20.8%) | 6  (12.5%) | 3  (6.3%) | ND |
|  | CBEm | 42 | 11 | 2  (4.8%) | 7  (16.7%) | 2  (4.8%) | 0  (0.0%) | 0  (0.0%) | 0  (0.0%) | ND |
|  | TadDBE | 44 | 27 | 4  (9.1%) | 10  (22.7%) | 2  (4.6%) | 7  (15.9%) | 3  (6.8%) | 1  (2.3%) | ND |
|  | hyDBE | 39 | 32 | 2  (5.1%) | 5  (12.8%) | 8  (20.5%) | 10  (25.6%) | 3  (7.7%) | 4  (10.3%) | ND |
| 7 | ABE9 | 47 | 34 | 8  (17.0%) | 11  (23.4%) | 13  (27.7%) | 1  (2.1%) | 1  (2.1%) | 0  (0.0%) | 0  (0.0%) |
|  | CBEm | 45 | 29 | 2  (4.4%) | 7  (15.6%) | 11  (24.4%) | 6  (13.3%) | 1  (2.2%) | 2  (4.4%) | 0  (0.0%) |
|  | TadDBE | 42 | 22 | 2  (4.8%) | 4  (9.5%) | 6  (14.3%) | 7  (16.7%) | 1  (2.4%) | 2  (4.8%) | 0  (0.0%) |
|  | hyDBE | 48 | 40 | 0  (0.0%) | 5  (10.4%) | 10  (20.8%) | 12  (25.0%) | 7  (14.6%) | 4  (8.3%) | 2  (4.2%) |

Note: mutated calli harboring any type of mutation at target sites were classified and statistically analyzed according to number of editing target sites. Proportion of multi-target sites edited simultaneously by each editor using 6 and 7 sgRNA cassettes were showed in parentheses, calculated by number of mutated calli with multiplex genome editing divided by total number of tested calli. ND, not detectable.

**Table S3.** The number of analyzed calli and mutation types at each target site showed in Figure 2D.

| Target NO. | BEs | Total number of tested calli | Number of mutated calli | Number of each mutation type | | |
| --- | --- | --- | --- | --- | --- | --- |
|  |  |  |  | Ho | He | Bi |
| *bsr-k1*-TS1 | ABE9 | 48 | 19 | 12 | 7 | 0 |
|  | CBEm | 42 | 11 | 4 | 7 | 0 |
|  | TadDBE | 44 | 23 | 9 | 6 | 8 |
|  | hyDBE | 39 | 18 | 4 | 11 | 3 |
| *IPI1*-TS2 | ABE9 | 48 | 21 | 13 | 8 | 0 |
|  | CBEm | 42 | 0 | 0 | 0 | 0 |
|  | TadDBE | 44 | 4 | 2 | 2 | 0 |
|  | hyDBE | 39 | 13 | 4 | 8 | 1 |
| *TubA2*-TS3 | ABE9 | 47 | 15 | 10 | 5 | 0 |
|  | CBEm | 45 | 10 | 4 | 6 | 0 |
|  | TadDBE | 42 | 14 | 6 | 3 | 5 |
|  | hyDBE | 48 | 8 | 2 | 4 | 2 |
| *ACCase*-TS4 | ABE9 | 47 | 2 | 0 | 2 | 0 |
|  | CBEm | 45 | 14 | 4 | 2 | 8 |
|  | TadDBE | 42 | 6 | 3 | 1 | 2 |
|  | hyDBE | 48 | 36 | 8 | 19 | 9 |
| *EPSPS*-TS5 | ABE9 | 47 | 1 | 0 | 1 | 0 |
|  | CBEm | 45 | 17 | 5 | 2 | 10 |
|  | TadDBE | 42 | 3 | 1 | 0 | 2 |
|  | hyDBE | 48 | 20 | 5 | 11 | 4 |
| *TT3.2*-TS6 | ABE9 | 48 | 11 | 8 | 3 | 0 |
|  | CBEm | 42 | 2 | 0 | 2 | 0 |
|  | TadDBE | 44 | 11 | 5 | 1 | 5 |
|  | hyDBE | 39 | 28 | 8 | 17 | 3 |
| *NRT1.1B*-TS7 | ABE9 | 47 | 18 | 14 | 4 | 0 |
|  | CBEm | 45 | 5 | 1 | 4 | 0 |
|  | TadDBE | 42 | 3 | 1 | 1 | 1 |
|  | hyDBE | 48 | 7 | 0 | 6 | 1 |
| *CPK4*-TS8 | ABE9 | 48 | 18 | 13 | 5 | 0 |
|  | CBEm | 42 | 0 | 0 | 0 | 0 |
|  | TadDBE | 44 | 13 | 6 | 3 | 4 |
|  | hyDBE | 39 | 22 | 5 | 14 | 3 |
| *TT3.1*-TS9 | ABE9 | 47 | 0 | 0 | 0 | 0 |
|  | CBEm | 45 | 6 | 2 | 4 | 0 |
|  | TadDBE | 42 | 0 | 0 | 0 | 0 |
|  | hyDBE | 48 | 5 | 1 | 3 | 1 |
| *CPK18*-TS10 | ABE9 | 48 | 28 | 7 | 2 | 19 |
|  | CBEm | 42 | 0 | 0 | 0 | 0 |
|  | TadDBE | 44 | 27 | 15 | 6 | 6 |
|  | hyDBE | 39 | 18 | 4 | 11 | 3 |
| *bsr-d1*-TS11 | ABE9 | 48 | 11 | 4 | 2 | 5 |
|  | CBEm | 42 | 9 | 3 | 6 | 0 |
|  | TadDBE | 44 | 1 | 0 | 1 | 0 |
|  | hyDBE | 39 | 12 | 4 | 7 | 1 |
| *PAY1*-TS12 | ABE9 | 47 | 0 | 0 | 0 | 0 |
|  | CBEm | 45 | 3 | 0 | 0 | 3 |
|  | TadDBE | 42 | 4 | 1 | 2 | 1 |
|  | hyDBE | 48 | 6 | 2 | 3 | 1 |
| *CPK4*-TS13 | ABE9 | 47 | 12 | 8 | 2 | 2 |
|  | CBEm | 45 | 11 | 4 | 5 | 2 |
|  | TadDBE | 42 | 12 | 4 | 4 | 4 |
|  | hyDBE | 48 | 14 | 1 | 8 | 5 |
| Total tested target sites | ABE9 | 617 | 156 | 89 (57%) | 41 (26%) | 26 (17%) |
|  | CBEm | 567 | 88 | 27 (30%) | 38 (43%) | 23 (27%) |
|  | TadDBE | 558 | 121 | 53 (44%) | 30 (25%) | 38 (31%) |
|  | hyDBE | 570 | 207 | 48 (23%) | 122 (59%) | 37 (18%) |

Note: mutated calli harboring any type of mutation at each target site were classified and statistically analyzed according to homozygous (Ho), heterozygous (He), and biallelic (Bi) mutation types. Average percentages of each mutation type induced by each BEs were showed in parentheses, calculated by number of each mutation type divided by number of mutated calli for TS1–TS13.

**Table S4.** Nucleotide substitutions, amino acid changes and editing frequencies induced by TadDBE at *OsBadh2* in T_0_ plants.

| Target | Target sequence (5′–3′) | Amino acid mutation | Frequency (%) |
| --- | --- | --- | --- |
| *Badh2*-TS14 | CAGG[AAC]TATCCTCTCCTGATGG | WT |  |
|  | CAGG[AGC]TATCCTCTCCTGATGG | N162S | 22.7 |
| *Badh2*-TS15 | GGACCAATGGCCAGATT[TGC]AGT | WT |  |
|  | GGACCAATGGCCAGATT[CGC]AGT | C294R | 40.9 |
|  | GGACCAATGGCCAGATT[CAC]AGT | C294H | 18.2 |
| *Badh2*-TS16 | ATTTCTACTGCAGGAAC[TAT]CCT | WT |  |
|  | ATTTCTACTGCAGGAAC[CAT]CCT | Y163H | 35.7 |
|  | ATTTCTACTGCAGGAAC[TAC]CCT | WT | 3.6 |
| *Badh2*-TS17 | CTCTCCTGATGGCAACA[TGG]AAG | WT |  |
|  | CTCTCCTGATGGCAACA[CAG]AAG | W170Q | 10.7 |
|  | CTCTCCTGATGGCAACA[CGG]AAG | W170R | 28.6 |
| *Badh2*-TS18 | CAGGAACTATCCTCTC[CTG]ATGG | WT |  |
|  | CAGGAACTATCCTCTC[CCG]ATGG | L166P | 44.0 |
| *Badh2*-TS19 | TTGACAGCCTGTTTCA[CTG][GAA]C | WT |  |
|  | TTGACAGCCTGTTTCA[CTG][AAA]C | E260K | 4.0 |
|  | TTGACAGCCTGTTTCA[CCG][GAA]C | L259P | 20.0 |
|  | TTGACAGCCTGTTTCA[CCA][AAA]C | L259P, E260K | 4.0 |
| *Badh2*-TS20 | TGAACTGCTCGCAACCC[TGC]TTC | WT |  |
|  | TGAACTGCTCGCAACCC[CGC]TTC | C453R | 50.0 |
|  | TGAACTGCTCGCAACCC[CAC]TTC | C453H | 10.0 |
| *Badh2*-TS21 | GCTTCTGCCAAGCTCCA[TGG]GGC | WT |  |
|  | GCTTCTGCCAAGCTCCA[CGG]GGC | W459R | 40.0 |
|  | GCTTCTGCCAAGCTCCA[TAG]GGC | W459* | 10.0 |
|  | GCTTCTGCCAAGCTCCA[CAG]GGC | W459Q | 10.0 |
|  | GCTTCTGCCAAGCTCCA[TAA]GGC | W459* | 5.0 |
| *Badh2*-TS22 | GACG[GAG]TACGCCTCCGATGAGC | WT |  |
|  | GACG[GGG]TACGCCTCCGATGAGC | E487G | 8.3 |
| *Badh2*-TS23 | GTAC[AAA]TCCCCTTCCAAGCTGT | WT |  |
|  | GTAC[AGA]TCCCCTTCCAAGCTGT | K498R | 6.3 |
| *Badh2*-TS24 | GTGACGGAGTACGCCTCC[GAT]GA | WT |  |
|  | GTGACGGAGTACGCCTCC[AAT]GA | D491N | 28.6 |
| *Badh2*-TS25 | TTCC[AAG][CTG]TAATGTAATATGC | WT |  |
|  | TTCC[GAG][CTG]TAATGTAATATGC | K502E | 42.9 |
|  | TTCC[AAG][TTG]TAATGTAATATGC | WT | 7.1 |
| *Badh2*-TS26 | ACGGAGTACGCC[TCC][GAT][GAG]CC | WT |  |
|  | ACGGAGTACGCC[TCC][GAC][GAG]CC | WT | 57.7 |
|  | ACGGAGTACGCC[TCC][GAT][AAG]CC | E492K | 7.7 |
|  | ACGGAGTACGCC[TCC][AAT][GAG]CC | D491N | 3.9 |
|  | ACGGAGTACGCC[TCC][GAC][AAG]CC | E492K | 3.9 |
|  | ACGGAGTACGCC[CCC][GAC][GAG]CC | S490P | 3.9 |
| *Badh2*-TS27 | GG[TAC][AAA]TCCCCTTCCAAGCTG | WT |  |
|  | GG[TGC][GAA]TCCCCTTCCAAGCTG | Y497C, K498E | 15.4 |
|  | GG[TGC][GGA]TCCCCTTCCAAGCTG | Y497C, K498G | 7.7 |

**Table S5.** Comparison of TadA-derived editors to other recently reported base editors.

| Base editors | | Function | Deaminases | | Nucleases | PAM | Average efficiency | Active windows | Tested species | Reference |
| --- | --- | --- | --- | --- | --- | --- | --- | --- | --- | --- |
| ABE | SpRY-ABE8e | A-to-G | TadA8e | | SpRYn | NHN (H = A/T/C) | 42.1% | A_4_–A_11_ | T_0_ rice | [1] |
|  | NRRH-ABE8e | A-to-G | TadA8e | | SpCas9n-NRRH | NRRH (R = A/G) | 84.0% | A_5_–A_8_ | T_0_ rice | [2] |
|  | NRCH-ABE8e |  |  |  | SpCas9n-NRCH | NRCH | 92.4% | A_4_–A_11_ |  |  |
|  | NRTH-ABE8e |  |  |  | SpCas9n-NRTH | NRTH | 100.0% | A_5_–A_11_ |  |  |
|  | SpG-ABE8e |  |  |  | SpGn | NGN | 91.1% | A_4_–A_7_ |  |  |
|  | SpRY-ABE8e |  |  |  | SpRYn | NNN | 15.4% | A_4_–A_7_ |  |  |
|  | rBE62 | A-to-G | TadA8e | | SpRYn | NAN | 62.1% | A_3_–A_6_ | T_0_ rice | [3] |
|  | rBE46b | A-to-G | TadA8e | | SpCas9n | NGG | 68.8% | A_4_–A_10_ | T_0_ rice | [4] |
|  | rBE50 |  |  |  | SpCas9n-NG | NGN | 73.4% | A_5_–A_8_ |  |  |
|  | rBE54 |  |  |  | ScCas9n | NNG | 52.1% | A_4_–A_11_ |  |  |
|  | rBE49b |  | TadA9 | | SpCas9n | NGG | 83.0% | A_1_–A_12_ |  |  |
|  | rBE53 |  |  |  | SpCas9n-NG | NGN | 89.1% | A_4_–A_10_ |  |  |
|  | rBE65 |  |  |  | SpRYn | NAN | 62.5% | A_3_–A_12_ |  |  |
|  | rBE57 |  |  |  | ScCas9n | NNG | 48.1% | A_3_–A_12_ |  |  |
|  | hyABE8e-NG | A-to-G | TadA8e | | SpCas9n-NG (DBD) | NGN | 67.8% | A_1_–A_13_ | T_0_ rice | [5] |
|  | hyABE8e-SpG |  |  |  | SpGn (DBD) | NGN | 73.9% | A_3_–A_14_ |  |  |
|  | hyABE8e-SpRY |  |  |  | SpRYn (DBD) | NNN | 74.7% | A_1_–A_13_ |  |  |
|  | PABE9e | A-to-G | TadA9e | | SpCas9n-NG | NGN | 60% | A_6_–A_9_ | Rice | [6] |
|  | TadABE9-SpRYn | A-to-G | TadA9 | | SpRYn | NNN | 25.30% | Main A_5_–A_6_ | Rice calli and T_0_ rice | In this study |
| CBE | Target-AID | C-to-T | PmCDA1 | | SpCas9n-NG | NGN | 37.7% | C_2_–C_12_ | T_0_ rice | [7] |
|  | A3Bctd-VHM-BE3 | C-to-T | A3Bctd-VHM | | nSaCas9 | NNGRRT | 23.1% | Single or double C-to-T edits | Rice protoplasts | [8] |
|  | A3Bctd-KKR-BE3 |  | A3Bctd-KKR | |  |  | 19.3% |  |  |  |
|  | BE4max/sCBE | C-to-T | rAPOBEC1 | | SpCas9n | NGG | 11.7% | C_4_–C_8_ | T_0_ rice | [9] |
|  | FNLS-sCBE |  |  |  |  |  | 17.5% |  |  |  |
|  | F4NLS-sCBE |  |  |  |  |  | 24.2% |  |  |  |
|  | Target-AID/DisSUGs | C-to-T | PmCDA1 | | SpCas9n | NGG | 66.0% | C_2_–C_12_ | T_0_ rice | [10] |
|  | CyDENT | C-to-T | hAPOBEC3A | | TALE & FokI | − | 20% in mitochondria | C_1_–C_18_ | Rice protoplasts | [11] |
|  | PhA3Amax-NG | C-to-T | hAPOBEC3A | | SpCas9n-NG | NGN | 39.5% | C_3_–C_13_ | T_0_ rice | [12] |
|  | PevorAC1-NG |  | evorAPOBEC1 | |  |  | 33.6% | C_3_–C_10_ |  |  |
|  | PevoFERNY-NG |  | evoFERNY | |  |  | 62.6% | C_3_–C_10_ |  |  |
|  | PevoCDA1-NG |  | evoPmCDA1 | |  |  | 27.6% | C_-1_–C_12_ |  |  |
|  | PevoCDA1-eNG |  | evoPmCDA1 | | SpCas9n-eNG |  | 40.1% | C_-1_–C_12_ |  |  |
|  | TadCBEa | C-to-T | TadCBEa | | nCas9-NG | NGN | 30.0% | C_4_–C_8_ | Rice protoplasts and T_0_ rice | [13] |
|  | TadCBEd |  | TadCBEd | |  |  | 27.6% |  |  |  |
|  | TadCBEd_V106W |  | TadCBEd_V106W | |  |  | 29.4% |  |  |  |
|  | rBE111a | C-to-T | TadA-CDd | | SpRYn | NNN | 57.4% | C_4_–C_8_ | T_0_ rice | [14] |
|  | rBE111b |  | TadA-E27R/N46L | |  |  | 43.6% |  |  |  |
|  | TadCBEm-SpRYn | C-to-D (D = T/A/G) | TadA-LM | | SpRYn | NNN | 15.50% | Main C_5_–C_6_ | Rice calli and T_0_ rice | In this study |
| DBE | STEMEs | C-to-T & A-to-G | APOBEC3A | ecTadA-TadA7.10 | SpCas9n | NGG | 13.2% | C_1_–C_17_ &  A_4_–A_8_ | T_0_ rice | [15] |
|  | STEME-NG |  |  |  | SpCas9n-NG | NGN |  |  |  |  |
|  | SWISS | C-to-T & A-to-G | APOBEC1 | ecTadA-TadA7.10 | SpCas9n-NG | NGN | 25.5% C-to-T, 16.4% A-to-G, 52.7% indels | − | Rice protoplasts | [16] |
|  | pDuBE1 | C-to-T & A-to-G | LjCDA1L-4 | TadA8e | SpCas9n | NGG | 49.7% | − | Rice protoplasts | [3] |
|  | hyA & C-Bemax | C-to-T & A-to-G | hAID | TadA8e | SpCas9n (DBD) | NGG | 33.2% | C_1_–C_15_ &  A_3_–A_9_ | HEK293T cells | [17] |
|  | MoBE | C-to-T & A-to-G | CDA1 | TadA9 | SpCas9n | NGG | 26.4% | − | T_0_ rice | [18] |
|  | STCBE-2 | C-to-T & A-to-G | evoFERNY | TadA8e | SpCas9n-NG | NGN | 23.1% for C and 24.2% for A, respectively | C_1_–C_14_ &  A_1_–A_10_ | Rice protoplasts | [19] |
|  | C-A-D-SpGn | C-to-T & A-to-G | evoFERNY | TadA8e | SpGn (DBD) | NGN | 41.90% | C_5_–C_9_ & A_3_, A_5_–A_9_ | T_0_ rice | [20] |
|  | TadDE | C-to-T & A-to-G | TadA-dual | | nCas9-NG | NGN | 15.9% | C_4_–C_8_ & A_4_–A_8_ | Rice protoplasts and T_0_ rice | [13] |
|  | rBE114a | C-to-T & A-to-G | TadA-dual | | SpCas9n | NGG | 75.0% | C_4_–C_8_ & A_4_–A_8_ | T_0_ rice | [14] |
|  | TadDBE-SpRYn | C-to-T & A-to-G | TadA-dual | | SpRYn | NNN | 11.0% for C and 20.0% for A, respectively | Main C_4_–C_5_ & A_4_–A_6_ | Rice calli and T_0_ rice | In this study |
|  | hyDBE-SpRYn |  | evoFERNY | TadA8e |  |  | 20.2% for C and 18.3% for A, respectively | C_3_–C_10_  & A_3_–A_11_ |  |  |

**Table S6.** Oligos used in this study.

| Oligos | Sequence (5′-3′) | Purpose |
| --- | --- | --- |
| *bsr-k1*-TS1 | CGTGCAGGGCGTCTTCCTATGGG | sgRNA sequence of the *bsr-k1*-TS1 for CRISPR-Skip. |
| *IPI1*-TS2 | CGGGATGGGCGCCGAGGAGGAGG | sgRNA sequence of the *IPI1*-TS2 for CRISPR-Silence. |
| *TubA2*-TS3 | AAAGCATGAAGTGGATCCTCGGG | sgRNA sequence of the *TubA2*-TS3 for saturation mutagenesis. |
| *ACCase*-TS4 | GAAACCACACTTGTCCAGCACGA | sgRNA sequence of the *ACCase*-TS4 for saturation mutagenesis. |
| *EPSPS*-TS5 | GCGACCATTGACAGCAGCCGTGA | sgRNA sequence of the *EPSPS*-TS5 for saturation mutagenesis. |
| *TT3.2*-TS6 | TGGCATGGCCTCCTCGCTGGCGT | sgRNA sequence of the *TT3.2*-TS6 for CRISPR-Silence. |
| *NRT1.1B*-TS7 | GGCGACGGCGAGCAAGTGGAGGC | sgRNA sequence of the *NRT1.1B*-TS7 for SNP substitutions. |
| *CPK4*-TS8 | TCCCAGTCCAAGAGGACCTCCAA | sgRNA sequence of the *CPK4*-TS8 for SNP substitutions. |
| *TT3.1*-TS9 | TCTTGCTGACAATTTCAGGTAAT | sgRNA sequence of the *TT3.1*-TS9 for SNP substitutions. |
| *CPK18*-TS10 | TACAAACTCCGAGGAGTGTTCAC | sgRNA sequence of the *CPK18*-TS10 for SNP substitutions. |
| *bsr-d1*-TS11 | TTTAACTGATAAATATAAGTATA | sgRNA sequence of the *bsr-d1*-TS11 for SNP substitutions. |
| *PAY1*-TS12 | TCCCCCGATTGGCTGTGCCACCC | sgRNA sequence of the *PAY1*-TS12 for SNP substitutions. |
| *CPK4*-TS13 | AGTCCAAGAGGACCTCCAAATCC | sgRNA sequence of the *CPK4*-TS13 for SNP substitutions. |
| *Badh2*-TS14 | CAGGAACTATCCTCTCCTGATGG | sgRNA sequence of the *Badh2*-TS14 for N162 mutation. |
| *Badh2*-TS15 | ACTGCAAATCTGGCCATTGGTCC | sgRNA sequence of the *Badh2*-TS15 for C294 mutation. |
| *Badh2*-TS16 | AGGATAGTTCCTGCAGTAGAAAT | sgRNA sequence of the *Badh2*-TS16 for Y163 mutation. |
| *Badh2*-TS17 | CTTCCATGTTGCCATCAGGAGAG | sgRNA sequence of the *Badh2*-TS17 for W170 mutation. |
| *Badh2*-TS18 | CCATCAGGAGAGGATAGTTCCTG | sgRNA sequence of the *Badh2*-TS18 for L166 mutation. |
| *Badh2*-TS19 | GTTCCAGTGAAACAGGCTGTCAA | sgRNA sequence of the *Badh2*-TS19 for E260 mutation. |
| *Badh2*-TS20 | GAAGCAGGGTTGCGAGCAGTTCA | sgRNA sequence of the *Badh2*-TS20 for C453 mutation. |
| *Badh2*-TS21 | GCCCCATGGAGCTTGGCAGAAGC | sgRNA sequence of the *Badh2*-TS21 for W459 mutation. |
| *Badh2*-TS22 | GACGGAGTACGCCTCCGATGAGC | sgRNA sequence of the *Badh2*-TS22 for E487 mutation. |
| *Badh2*-TS23 | GTACAAATCCCCTTCCAAGCTGT | sgRNA sequence of the *Badh2*-TS23 for K498 mutation. |
| *Badh2*-TS24 | TCATCGGAGGCGTACTCCGTCAC | sgRNA sequence of the *Badh2*-TS24 for D491 mutation. |
| *Badh2*-TS25 | TTCCAAGCTGTAATGTAATATGC | sgRNA sequence of the *Badh2*-TS25 for K502 mutation. |
| *Badh2*-TS26 | GGCTCATCGGAGGCGTACTCCGT | sgRNA sequence of the *Badh2*-TS26 for D491, E492 mutations. |
| *Badh2*-TS27 | GGTACAAATCCCCTTCCAAGCTG | sgRNA sequence of the *Badh2*-TS27 for K498 mutation. |
| *Badh2*-KO | CGCGATTGCGCGGAGGTACTTGG | sgRNA sequence for *Badh2* knockout. |
| F-on-TS1  R-on-TS1 | cggagtgagcacCTCCTCCATTGCGGGCTTAT  gaggctggatggGCTGATAGCACTTGGCCGCT | Amplifying the on-target fragment of *bsr-k1*-TS1 site for NGS analysis. |
| F-on-TS2  R-on-TS2 | cggagtgagcacGGTGGGCGCGTGTTCTTCGT  gaggctggatggAGGCAGATCGAGCACGGGAC | Amplifying the on-target fragment of *IPI1*-TS2 site for NGS analysis. |
| F-on-TS3  R-on-TS3 | cggagtgagcacAATGCTAATGTGCTGCAGGT  gaggctggatggAAGGCGCTGTTGGTGATCT | Amplifying the on-target fragment of *TubA2*-TS3 site for NGS analysis. |
| F-on-TS4  R-on-TS4 | cggagtgagcacAAGCTTGGTGGAATTCCAGT  gaggctggatggTAGCGAGGATGAACAGAGGT | Amplifying the on-target fragment of *ACCase*-TS4 site for NGS analysis. |
| F-on-TS5  R-on-TS5 | cggagtgagcacTGCAAAAAGAGCTGTAGTCG  gaggctggatggATACCCCATGAATTCCATAC | Amplifying the on-target fragment of *EPSPS*-TS5 site for NGS analysis. |
| F-on-TS6  R-on-TS6 | cggagtgagcacACTACTACGTACTGGTCGCT  gaggctggatggGGATCCTCGAGCGCCGAGCA | Amplifying the on-target fragment of *TT3.2*-TS6 site for NGS analysis. |
| F-on-TS7  R-on-TS7 | cggagtgagcacACCGTTAGAACCACCAAGGT  gaggctggatggGCGTGGATGGTCCAGAACAT | Amplifying the on-target fragment of *NRT1.1B*-TS7 site for NGS analysis. |
| F-on-TS8  R-on-TS8 | cggagtgagcacGGTTCCATCGAGCCATTGCT  gaggctggatggGGACAGGAACAAGACAACCT | Amplifying the on-target fragment of *CPK4*-TS8 site for NGS analysis. |
| F-on-TS9  R-on-TS9 | cggagtgagcacAAAAGTAAGCCGACTGTTGA  gaggctggatggAAGGCACATACAATCGTGCC | Amplifying the on-target fragment of *TT3.1*-TS9 site for NGS analysis. |
| F-on-TS10  R-on-TS10 | cggagtgagcacCAAACAGGACTGAAAGGTTC  gaggctggatggCAACAATGACTGCCCTTTGT | Amplifying the on-target fragment of *CPK18*-TS10 site for NGS analysis. |
| F-on-TS11  R-on-TS11 | cggagtgagcacGTGTTTTTAGCGATCTAAAATC  gaggctggatggGATGAACCTGTACTTTCTGT | Amplifying the on-target fragment of *bsr-d1*-TS11 site for NGS analysis. |
| F-on-TS12  R-on-TS12 | cggagtgagcacTGGGGAGAACCAACAAACAT  gaggctggatggAGCTCCAATAGATCCAGAAG | Amplifying the on-target fragment of *PAY1*-TS12 site for NGS analysis. |
| F-on-TS13  R-on-TS13 | cggagtgagcacATCGACAAAGACGGGAGAAT  gaggctggatggAACATCACATGGCACGCGGA | Amplifying the on-target fragment of *CPK4*-TS13 site for NGS analysis. |
| F-on-T14161718  R-on-T14161718 | cggagtgagcacGGGAAAACAATTGATAACGG  gaggctggatggAAACTTACACGGAAGCCAAT | Amplifying the on-target fragment of *Badh2*-TS14, TS16–TS18 site for NGS analysis. |
| F-on-TS15  R-on-TS15 | cggagtgagcacCTGGTTATGGACTCTGTTTG  gaggctggatggTGAACTCAGGAGTTGATCAG | Amplifying the on-target fragment of *Badh2*-TS15 site for NGS analysis. |
| F-on-TS19  R-on-TS19 | cggagtgagcacTCAATTCTCAATGTTGTCCT  gaggctggatggGTTAATCATAGCAAGTGGCA | Amplifying the on-target fragment of *Badh2*-TS19 site for NGS analysis. |
| F-on-TS2021  R-on-TS2021 | cggagtgagcacCCACATCATCTGATCCATGA  gaggctggatggGATTGTTGTGTGCTACCCAC | Amplifying the on-target fragment of *Badh2*-TS20, TS21 site for NGS analysis. |
| F-on-TS22-27  R-on-TS22-27 | cggagtgagcacTGATGATCTCGACCTGACAT  gaggctggatggCCGTCATACTATGGCCTCTT | Amplifying the on-target fragment of *Badh2*-TS22–TS27 site for NGS analysis. |
| F-self-OsU6a  F-self-OsU6b  F-self-OsU3  R-self-sgRNA | gaggctggatggAGGTGCTTACGTGCGAGGT  gaggctggatggGCCGACGAGCGTGTACTAC  gaggctggatggCCATGAAGCCTTTCAGGAC  cggagtgagcacCGGTGCCACTTTTTCAAGTTG | Amplifying the sgRNA cassette fragments for NGS analysis. |

Note: The underlines represent the PAM sequences. Small letters of the nucleotides are adapter bases for binding the barcoded primers used for NGS.

**Supplemental reference**

[1] Q. Ren, S. Sretenovic, S. Liu, X. Tang, L. Huang, Y. He, L. Liu, Y. Guo, Z. Zhong, G. Liu, Y. Cheng, X. Zheng, C. Pan, D. Yin, Y. Zhang, W. Li, L. Qi, C. Li, Y. Qi, Y. Zhang, PAM-less plant genome editing using a CRISPR-SpRY toolbox, *Nat. Plants* **2021**, *7*, 25–33.

[2] J. Li, R. Xu, R. Qin, X. Liu, F. Kong, P. Wei, Genome editing mediated by SpCas9 variants with broad non-canonical PAM compatibility in plants, *Mol. Plant* **2021**, *14*, 352–360.

[3] Z. Xu, Y. Kuang, B. Ren, D. Yan, F. Yan, C. Spetz, W. Sun, G. Wang, X. Zhou, H. Zhou, SpRY greatly expands the genome editing scope in rice with highly flexible PAM recognition, *Genome Biol*. **2021**, *22*, 6.

[4] D. Yan, B. Ren, L. Liu, F. Yan, S. Li, G. Wang, W. Sun, X. Zhou, H. Zhou, High-efficiency and multiplex adenine base editing in plants using new TadA variants, *Mol. Plant* **2021**, *14*, 722–731.

[5] J. Tan, D. Zeng, Y. Zhao, Y. Wang, T. Liu, S. Li, Y. Xue, Y. Luo, X. Xie, L. Chen, Y.G. Liu, Q. Zhu, PhieABEs: a PAM-less/free high-efficiency adenine base editor toolbox with wide target scope in plants, *Plant Biotechnol. J*. **2022**, *20*, 934–943.

[6] T. Tu, Z. Song, X. Liu, S. Wang, X. He, H. Xi, J. Wang, T. Yan, H. Chen, Z. Zhang, X. Lv, J. Lv, X. Huang, J. Zhao, C. Lin, C. Gao, J. Zhang, F. Gu, A precise and efficient adenine base editor, *Mol. Ther*. **2022**, *30*, 2933–2941.

[7] Z. Zhong, S. Sretenovic, Q. Ren, L. Yang, Y. Bao, C. Qi, M. Yuan, Y. He, S. Liu, X. Liu, J. Wang, L. Huang, Y. Wang, D. Baby, D. Wang, T. Zhang, Y. Qi, Y. Zhang, Improving plant genome editing with high-fidelity xCas9 and non-canonical PAM-targeting Cas9-NG, *Mol. Plant* **2019**, *12*, 1027–1036.

[8] S. Jin, H. Fei, Z. Zhu, Y. Luo, J. Liu, S. Gao, F. Zhang, Y. Chen, Y. Wang, C. Gao, Rationally designed APOBEC3B cytosine base editors with improved specificity, *Mol. Cell* **2020**, *79*, 728–740.

[9] F. Wang, C. Zhang, W. Xu, S. Yuan, J. Song, L. Li, J. Zhao, J. Yang, Developing high-efficiency base editors by combining optimized synergistic core components with new types of nuclear localization signal peptide, *Crop J*. **2020**, *8*, 408–417.

[10] W. Xu, Y. Yang, Y. Liu, G. Kang, F. Wang, L. Li, X. Lv, S. Zhao, S. Yuan, J. Song, Y. Wu, F. Feng, X. He, C. Zhang, W. Song, J. Zhao, J. Yang, Discriminated sgRNAs-based surrogate system greatly enhances the screening efficiency of plant base-edited cells, *Mol. Plant*, **2020**, *13*, 169–180.

[11] J. Hu, Y. Sun, B. Li, Z. Liu, Z. Wang, Q. Gao, M. Guo, G. Liu, K. Zhao, C. Gao, Strand-preferred base editing of organellar and nuclear genomes using CyDENT, *Nat. Biotechnol*. **2024**, *42*, 936–945.

[12] D. Zeng, T. Liu, J. Tan, Y. Zhang, Z. Zheng, B. Wang, D. Zhou, X. Xie, M. Guo, Y.G. Liu, Q. Zhu, PhieCBEs: plant high-efficiency cytidine base editors with expanded target range, *Mol. Plant* **2020**, *13*, 1666–1669.

[13] T. Fan, Y. Cheng, Y. Wu, S. Liu, X. Tang, Y. He, S. Liao, X. Zheng, T. Zhang, Y. Qi, Y. Zhang, High performance TadA-8e derived cytosine and dual base editors with undetectable off-target effects in plants, *Nat. Commun*. **2024**, *15*, 5103.

[14] M. Yu, Y. Kuang, C. Wang, X. Wu, S. Li, D. Zhang, W. Sun, X. Zhou, B. Ren, H. Zhou, Diverse nucleotide substitutions in rice base editing mediated by novel TadA variants, *Plant Commun*. **2024**, *5*, 100926.

[15] C. Li, R. Zhang, X. Meng, S. Chen, Y. Zong, C. Lu, J. Qiu, Y. Chen, J. Li, C. Gao, Targeted, random mutagenesis of plant genes with dual cytosine and adenine base editors, *Nat. Biotechnol*. **2020**, *38*, 875–882.

[16] C. Li, Y. Zong, S. Jin, H. Zhu, D. Lin, S. Li, J. Qiu, Y. Wang, C. Gao, SWISS: multiplexed orthogonal genome editing in plants with a Cas9 nickase and engineered CRISPR RNA scaffolds, *Genome Biol*. **2020**, *21*, 141.

[17] N. Xue, X. Liu, D. Zhang, Y. Wu, Y. Zhong, J. Wang, W. Fan, H. Jiang, B. Zhu, X. Ge, R.V. Gonzalez, L. Chen, S. Zhang, P. She, Z. Zhong, J. Sun, X. Chen, L. Wang, Z. Gu, P. Zhu, M. Liu, D. Li, T. Zhong, X. Zhang, Improving adenine and dual base editors through introduction of TadA-8e and Rad51DBD, *Nat. Commun*. **2023**, *14*, 1224.

[18] A. Zhang, T. Shan, Y. Sun, Z. Chen, J. Hu, Z. Hu, Z. Ming, Z. Zhu, X. Li, J. He, S. Liu, L. Jiang, X. Dong, Y. Wu, Y. Wang, Y. Liu, C. Li, J. Wan, Directed evolution rice genes with randomly multiplexed sgRNAs assembly of base editors, *Plant Biotechnol. J*, **2023**, *21*, 2597–2610.

[19] C. Zhang, X. Zhong, S. Li, L. Yan, J. Li, Y. He, Y. Lin, Y. Zhang, L. Xia, Artificial evolution of OsEPSPS through an improved dual cytosine and adenine base editor generated a novel allele conferring rice glyphosate tolerance, *J. Integr. Plant Biol*. **2023**, *65*, 2194–2203.

[20] Z. Zheng, T. Liu, N. Chai, D. Zeng, R. Zhang, Y. Wu, J. Hang, Y. Liu, Q. Deng, J. Tan, J. Liu, X. Xie, Y.G. Liu, Q. Zhu, PhieDBEs: a DBD-containing, PAM-flexible, high-efficiency dual base editor toolbox with wide targeting scope for use in plants, *Plant Biotechnol. J*. **2024**, *22*, 3164–3174.
